# Multi-omics reveals molecular signatures of moderate intensity exercise and identifies candidate exercise mimetics in mice

**DOI:** 10.1016/j.redox.2026.104186

**Published:** 2026-04-22

**Authors:** Hanlin Jiang, Shota Inoue, Junpei Hatakeyama, Hideki Moriyama

**Affiliations:** a Department of Rehabilitation Science, Graduate School of Health Sciences, Kobe University, Kobe, Japan; b Life and Medical Sciences Area, Health Sciences Discipline, Kobe University, Kobe, Japan

**Keywords:** exercise, skeletal muscle, Transcriptome, Phosphoproteome, Methylome, apigenin, doxazosin

## Abstract

Exercise is known to promote systemic health and prevent various chronic diseases. However, the molecular mechanisms underlying its beneficial effects remain incompletely understood. Although the health benefits of exercise have been widely studied, most research has treated exercise as a general intervention, without clearly standardizing its intensity. This study focused on a physiologically and molecularly defined moderate intensity, which may uniquely capture the core health-promoting mechanisms of exercise. To characterize the molecularly defined moderate intensity exercise, integrative multi-omics analyses—including transcriptomics, epigenomics, and phosphoproteomics—were performed using skeletal muscle tissue. These analyses revealed that this specifically defined exercise consistently modulated shared molecular pathways across both exercise modalities, especially insulin signaling, FoxO signaling, and circadian rhythm regulation. To explore the translational relevance of the identified molecular signatures, Connectivity Map analysis was used to search for compounds with similar transcriptional profiles. As a secondary outcome, apigenin and doxazosin were found to mimic exercise-associated molecular responses partially. These compounds exerted distinct physiological effects in vivo, including enhanced mitochondrial function and endurance and muscle hypertrophy with musculoskeletal protection. In conclusion, this study primarily elucidates the systemic molecular basis of physiologically and molecularly defined moderate-intensity exercise. The identification of candidate exercise mimetics serves as a potential application of these findings.

## Graphical abstract

**Figure:**
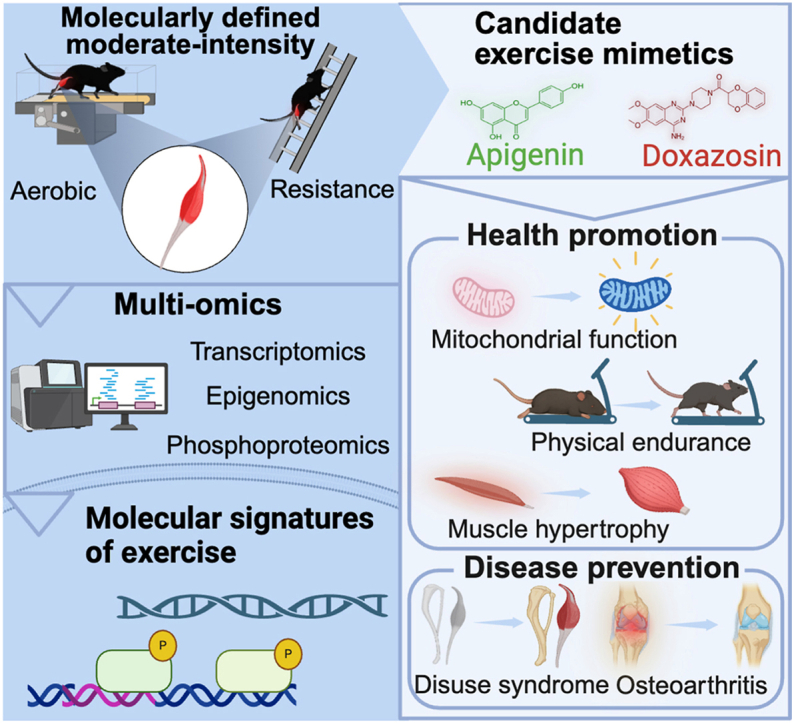

## 1 Introduction

Exercise significantly enhances overall health, impacting many parts of the body: the skeletal muscle, the cardiovascular system, the immune system, cranial nerves, bones, and abdominal organs [1]. There is growing evidence to support the efficacy of exercise in managing various conditions, including obesity, musculoskeletal disorders, cardiovascular disease, metabolic syndrome, and neurodegenerative disorders [2]. Consequently, exercise can be considered a genuine polypill owing to its nonpharmacological benefits for treating these conditions.

Among various forms of exercise, moderate-intensity exercise is most widely recommended for its safety, accessibility, and sustainability. This level of exercise is particularly effective in eliciting adaptive responses without provoking excessive physiological stress. Indeed, suboptimal intensity may fail to trigger beneficial adaptations, while excessive intensity can induce detrimental effects such as oxidative stress, mitochondrial dysfunction, and impaired protein homeostasis [3,4]. Despite its clinical relevance, the molecularly defined moderate intensity of exercise—defined as the intensity that maximizes benefits while minimizing harm—has not been consistently determined or validated in previous molecular studies.

Skeletal muscle is a primary effector organ of exercise, contributing to systemic adaptations via mechanical stress, metabolic remodeling, and myokine secretion [5,6]. While many studies have examined exercise-induced molecular changes in skeletal muscle [[7], [8], [9]], most have focused exclusively on aerobic training, lacked standardization in exercise protocols, or failed to control for exercise intensity. Notably, few have defined or validated an “optimal” intensity capable of eliciting robust and reproducible molecular responses [10,11]. In the present study, we focused on skeletal muscle as the largest organ of movement and an endocrine regulator that mediates cross-tissue benefits, thereby providing a rational basis for investigating systemic implications through muscle-centered multi-omics analyses. This gap has hindered efforts to establish consistent molecular signatures that underpin the health-promoting effects of physical activity.

To address these limitations, the present study was designed to: (1) experimentally define a working model of molecularly defined moderate intensities of aerobic and resistance exercise in mice based on physiological and molecular criteria, and (2) comprehensively characterize the molecular adaptations induced by these optimal intensities through multi-omics profiling—specifically transcriptomic, epigenomic, and phosphoproteomic analyses of skeletal muscle. By elucidating the integrated regulatory networks activated by optimal-intensity exercise, this study provides a mechanistic framework for understanding how moderate physical activity promotes systemic health.

As a secondary objective, we also investigated whether the molecular signatures of optimal exercise could inform the identification of candidate compounds that mimic these effects. Hereafter, we use the term exercise-mimetic candidates to denote pharmacological agents that reproduce selected molecular and physiological features of moderate exercise. These candidates may complement lifestyle interventions by partially recapitulating exercise-associated benefits, while not encompassing the full systemic effects of physical activity. To assess the translational relevance of such compounds, we employed disease models. Specifically, we tested their efficacy in hindlimb unloading (HLU, disuse-induced muscle and bone loss), destabilization of the medial meniscus (DMM, osteoarthritis), estrogen deficiency–induced osteoporosis (EDOP), high-fat diet (HFD)–induced obesity, and natural aging. Clinically, physical exercise has been demonstrated to ameliorate disuse-induced musculoskeletal decline [12], osteoarthritis progression [13], and osteoporosis [14], as well as to mitigate obesity- [15] and aging-related dysfunctions [16].

## 2 Results

### 2.1 Determining the moderate intensity of exercise that enhances muscle adaptation and mitochondrial function in mice

**Fig. 1:**
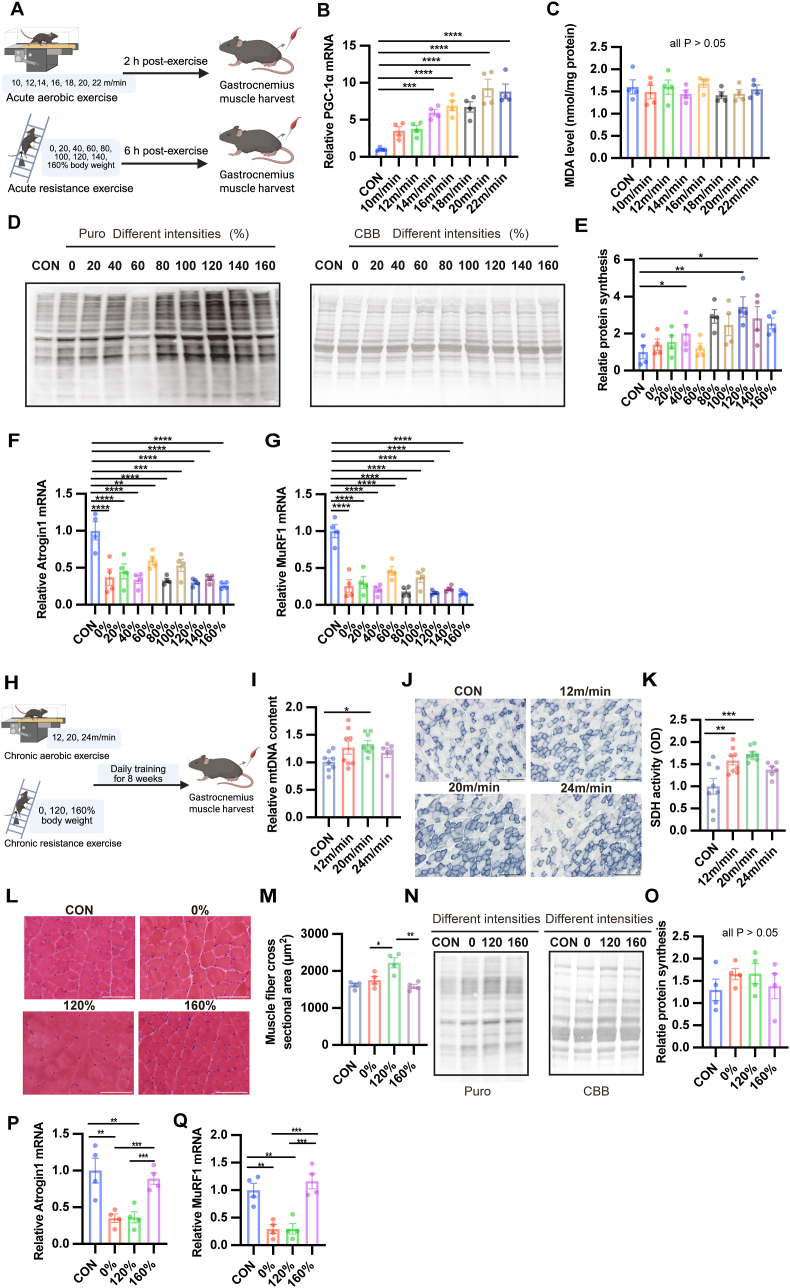
**Identification of moderate intensity of aerobic and resistance exercise through acute and chronic exercise.** (A) Schematic diagram of acute aerobic (treadmill, 10–22 m/min) and resistance (ladder climbing with 0–160% body weight) exercise. (B) Relative mRNA expression of PGC-1α in gastrocnemius muscle after acute aerobic exercise at different intensities. (C) MDA levels in the gastrocnemius muscle following acute aerobic exercise. (D) Representative images of puromycin-labeled peptides (SUnSET assay) and CBB staining after acute resistance exercise with varying intensities. (E) Quantification of relative protein synthesis based on puromycin incorporation. (F, G) Relative mRNA expression of Atrogin-1 (F) and MuRF-1 (G) following acute resistance exercise. (H) Schematic diagram of chronic aerobic (12, 20, or 24 m/min) and resistance (0, 120, or 160% body weight) exercise for 8 weeks, followed by muscle harvest. (I) Relative mtDNA content in gastrocnemius muscle after chronic aerobic exercise. (J) Representative images of SDH staining in the gastrocnemius after chronic aerobic exercise. Scale bars, 200 μm. (K) Quantification of SDH activity in the gastrocnemius muscle. (L) Representative images of hematoxylin and eosin staining after chronic resistance exercise with varying intensities. Scale bars, 100 μm. (M) Quantification of gastrocnemius muscle fiber cross-sectional area. (N) Representative images of puromycin-labeled peptides and CBB staining after chronic resistance exercise with varying intensities. (O) Quantification of relative protein synthesis. (P, Q) Relative mRNA expression levels of Atrogin-1 (P) and MuRF-1 (Q) after chronic resistance exercise. Data are presented as mean ± SEM. Statistical significance was determined using one-way ANOVA followed by Tukey's post hoc test. ∗P < 0.05, ∗∗P < 0.01, ∗∗∗P < 0.001, ∗∗∗∗P < 0.0001.

To identify the moderate intensity of exercise to achieve optimal skeletal muscle adaptation in mice, we subjected healthy young mice to various intensities of aerobic and resistance exercise and monitored key physiological markers (Fig. 1A). All mice assigned to the aerobic and resistance exercise groups successfully completed the prescribed training protocols. After the acute exercise, we observed an intensity-dependent increase in PGC-1α mRNA, a key regulator of mitochondrial biogenesis [17], with the peak increase observed when the treadmill speed was 20 m/min (Fig. 1B). MDA, which is a marker of lipid peroxidation, remained stable across all aerobic intensities (Fig. 1C). Furthermore, the mRNA expression of Sod1, a primary antioxidant enzyme, remained entirely stable across all tested aerobic and resistance intensities (Fig. S1). These findings collectively suggest that exercise at this molecularly defined moderate intensity maximizes adaptive signaling without exacerbating oxidative stress or forcing a compensatory depletion of the basal antioxidant defense system, resulting in no overt changes in these selected oxidative stress markers. In contrast, resistance exercise maximized muscle protein synthesis at 120% of body weight (Fig. 1D and E). Notably, the markers of protein degradation, Atrogin-1 and MuRF-1, were generally decreased by all exercise intensities (Fig. 1F and G), suggesting a universal anticatabolic effect of resistance exercise.

To assess the long-term efficacy of a single daily exercise session, mice underwent 8 weeks of aerobic or resistance training at various intensities (Fig. 1H). Aerobic exercise at 20 m/min significantly increased mitochondrial DNA content and SDH activity in skeletal muscle (Fig. 1I–K), whereas a higher intensity (24 m/min) failed to induce such changes, underscoring the threshold nature of moderate-intensity aerobic stimuli. Resistance exercise at 120% of body weight significantly promoted skeletal muscle hypertrophy (Fig. 1L and M), indicating that 120% of body weight was the most effective among the three tested intensities in promoting muscle hypertrophy. Although puromycin incorporation tended to increase following resistance training, particularly at 120%, no statistically significant differences were observed among the groups (Fig. 1N and O). At this intensity, reductions in Atrogin-1 and MuRF-1 occurred, an observation not replicated for 160% of body weight (Fig. 1P and Q). These findings demonstrate that intensity critically influences skeletal muscle adaptation and identify 20 m/min (aerobic) and 120% body mass (resistance) as population-level optimal moderate intensities under our standardized protocols—i.e., the group-prescribed loads that maximized adaptive markers without evidence of excessive stress. Although this initial phase served strictly as a molecular intensity screening, the functional efficacy of these selected moderate intensities—such as enhanced endurance and grip strength—was explicitly validated in our subsequent in vivo experiments (see Fig. 5, Fig. 6).

### 2.2 Gene expression changes in skeletal muscle following moderate exercise

**Fig. 2:**
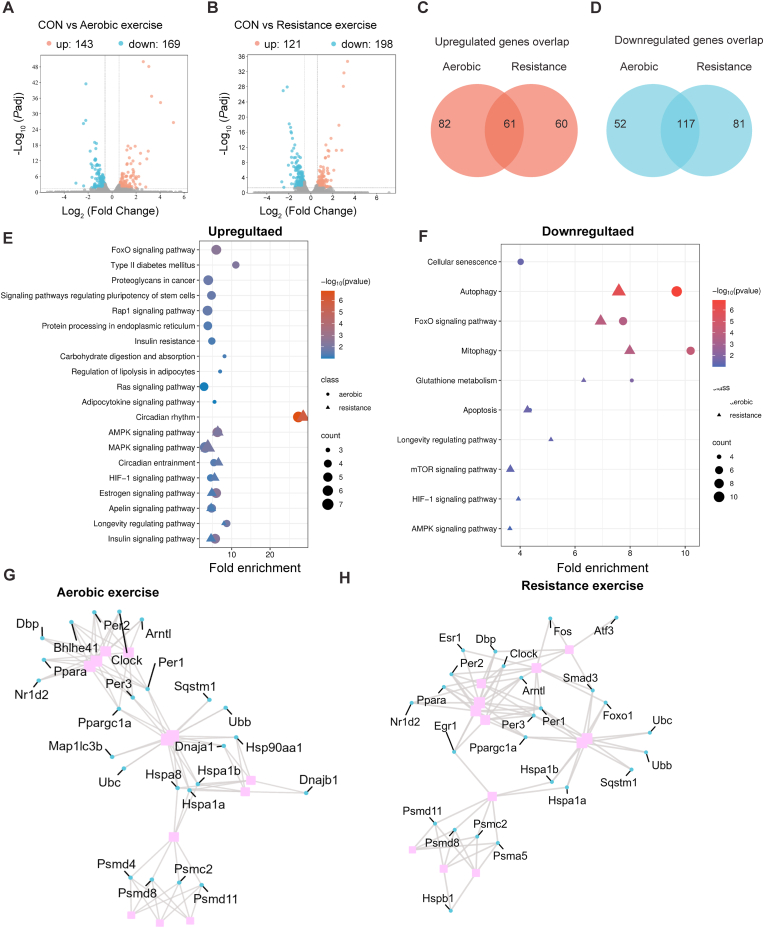
**Transcriptomic responses and pathway enrichment in skeletal muscle following moderate exercise.** (A, B) Volcano plots showing differentially expressed genes (DEGs) in gastrocnemius muscle after acute moderate aerobic (A) and resistance (B) exercise, as determined by RNA-seq. DEGs were defined by adjusted p-value (FDR) < 0.05. (C, D) Venn diagrams indicating the number of overlapping upregulated (C) and downregulated (D) genes shared between aerobic and resistance exercise groups. (E, F) KEGG pathway enrichment analysis of upregulated (E) and downregulated (F) genes in aerobic and resistance exercise groups. Bubble plots represent fold enrichment, p-values, and gene counts. KEGG pathway enrichment was performed using DAVID, with a significance threshold of p < 0.05. (G, H) PPI networks constructed from DEGs in aerobic (G) and resistance (H) exercise groups. Networks were generated using STRING with a minimum required interaction score >0.9, and hub genes were identified by degree centrality using the cytoHubba plugin in Cytoscape.

To explore the molecular responses of skeletal muscle to moderate exercise, we analyzed gene expression changes via RNA-seq following aerobic (20 m/min treadmill) and resistance (120% body weight ladder climb) exercises. Relative to the control (no exercise) group, aerobic exercise upregulated 143 genes and downregulated 169 genes, whereas resistance exercise upregulated 121 genes and downregulated 198 genes (Fig. 2A and B). Notably, 61 upregulated and 117 downregulated genes were common to both exercise modes, highlighting shared molecular adaptations (Fig. 2C and D). To understand the functional relevance of exercise-induced gene changes, we performed KEGG pathway enrichment analyses separately for upregulated and downregulated genes in aerobic and resistance exercise groups. As shown in Fig. 2E, circadian rhythm–related pathways were enriched among both upregulated and downregulated genes in both exercise modes, indicating transcriptional remodeling of the circadian system rather than unidirectional activation. Additional upregulated pathways included insulin signaling, AMPK signaling, and FoxO signaling, suggesting enhanced metabolic coordination and stress adaptation. Conversely, downregulated genes were enriched in pathways associated with autophagy, and FoxO signaling (Fig. 2F), which are typically upregulated in muscle atrophy, oxidative stress, or excessive loading. Their suppression under moderate exercise suggests a transition toward reduced proteolysis and cellular stress, favoring an anabolic and homeostatic environment. These findings reflect a fine-tuned balance between stress responses and protein turnover under moderate exercise.

The PPI networks revealed multiple hub genes shared across both exercise modalities (Fig. 2G and H). Notably, central nodes included genes associated with the ubiquitin–proteasome system (Psmd11, Psmd8, Ubb, Ubc), circadian rhythm (Clock, Per1/2, Arntl), and proteostasis-related heat shock proteins (Hspa1a, Hspa1b). These results demonstrate that moderate-intensity exercise, regardless of mode, induces convergent transcriptional programs that support circadian regulation, protein homeostasis, and cellular resilience in skeletal muscle.

### 2.3 Moderate exercise induces coordinated epigenetic and transcriptional regulation in skeletal muscle

**Fig. 3:**
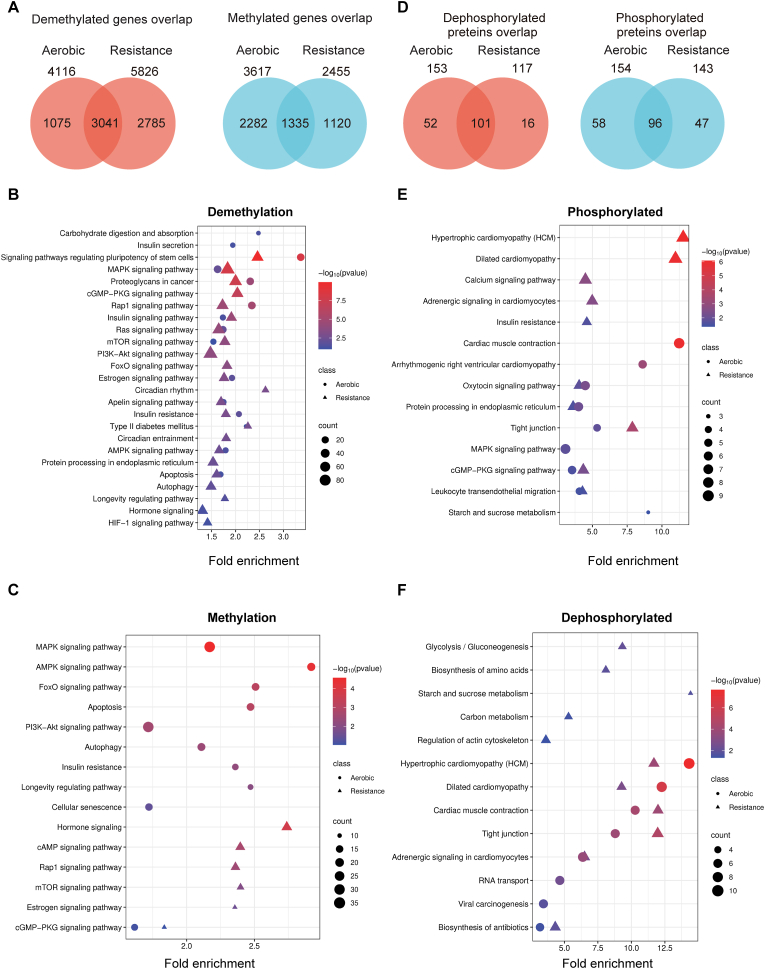
**Epigenomic and phosphoproteomic responses in skeletal muscle following moderate exercise.** (A) Venn diagrams showing the number of genes that were demethylated or methylated after moderate aerobic and resistance exercise, as identified by RRBS. Differentially methylated genes (DMGs) were defined as promoter CpG loci with FDR <0.05. (B, C) KEGG pathway enrichment analysis of demethylated (B) and methylated (C) genes following moderate exercise. (D) Venn diagrams showing the number of proteins with decreased or increased phosphorylation following moderate aerobic and resistance exercise, as identified by phosphoproteomic analysis using IBT. Differentially phosphorylated proteins were defined by fold change >1.2 or <0.833 with p < 0.05. (E, F) KEGG pathway enrichment analysis of phosphorylated (E) and dephosphorylated (F) proteins following moderate exercise. Pathway enrichment was performed using DAVID (v6.8), with a significance threshold of p < 0.05. Note: In bubble plots (B–C and E–F), bubble size indicates the number of genes or proteins per pathway; color intensity reflects statistical significance.

To assess epigenetic responses to moderate exercise, we performed RRBS on skeletal muscle after a single session of aerobic (20 m/min) or resistance (120% body weight) exercise. Both exercise modalities induced substantial DNA methylation changes, including 3041 genes commonly demethylated and 1335 commonly methylated between the two (Fig. 3A). Demethylated genes were enriched in key adaptive pathways, including MAPK, AMPK, insulin, and FoxO signaling, as well as synaptic and insulin resistance (Fig. 3B), consistent with upregulated expression of these same pathways in RNA-seq data (Fig. 2E). Conversely, methylated genes were enriched in autophagy, FoxO and apoptosis signaling (Fig. 3C), which were downregulated in transcriptomic data (Fig. 2F). These findings suggest that exercise promotes beneficial gene activation via demethylation while silencing potentially maladaptive pathways through methylation. Pathways such as FoxO and proteasome showed consistent changes across both omics’ layers, supporting a model of integrated epigenetic and transcriptional remodeling underlying exercise adaptation.

### 2.4 Phosphoproteomic changes support downstream pathway activation

To investigate downstream molecular consequences, we performed phosphoproteomic profiling. Overlapping analysis revealed 101 proteins dephosphorylated and 96 phosphorylated in both exercise modes (Fig. 3D). Pathway enrichment analysis revealed that both aerobic and resistance exercise significantly enhanced phosphorylation of enzymes involved in glycolysis and the pentose phosphate pathway, indicating a metabolic shift toward glycolytic and redox-supportive adaptations rather than oxidative phosphorylation (Fig. 3E). Additionally, increased phosphorylation was observed in the MAPK and cGMP–PKG signaling pathways, both of which are involved in protein synthesis regulation, vascular homeostasis, and neuromuscular signaling, suggesting anabolic and functional remodeling. In resistance exercise specifically, phosphorylation changes were enriched in the insulin resistance pathway, which are essential for mTORC1 activation and glucose metabolism. This suggests a load-specific activation of hypertrophic signaling. Conversely, dephosphorylation events were enriched in pathways associated with cardiac muscle contraction, hypertrophic cardiomyopathy, and dilated cardiomyopathy, especially under aerobic exercise (Fig. 3F). Although these pathways are primarily defined in cardiac tissue, their downregulation in skeletal muscle may reflect adaptive desensitization of mechanotransductive and structural signaling, potentially serving a protective role during repetitive contractions. Collectively, these phosphoproteomic changes indicate that moderate-intensity exercise induces a coordinated response in skeletal muscle, combining enhanced glycolytic flux and anabolic signaling with structural tuning of contractile systems.

Specifically, qPCR analysis confirmed the synchronized upregulation of core circadian rhythm genes (Per2, Bmal1, Rev-erbα) precisely at these defined moderate intensities (Fig. S2A–F). Correspondingly, the robust activation of major energy-sensing and mechanotransduction kinase cascades (*p*-AMPK, p-p38 MAPK, p-p70 S6K, and *p*-ERK1/2) was successfully confirmed via Western blotting across broad intensity gradients, exhibiting robust and synchronized responses at 20 m/min and 120% body weight (Fig. S3). This distinct validation phase provides robust independent biological confirmation for our hypothesis-generating multi-omics datasets.

### 2.5 Identification of potential exercise mimetics through connectivity analysis of post-exercise gene expression profiles

**Fig. 4:**
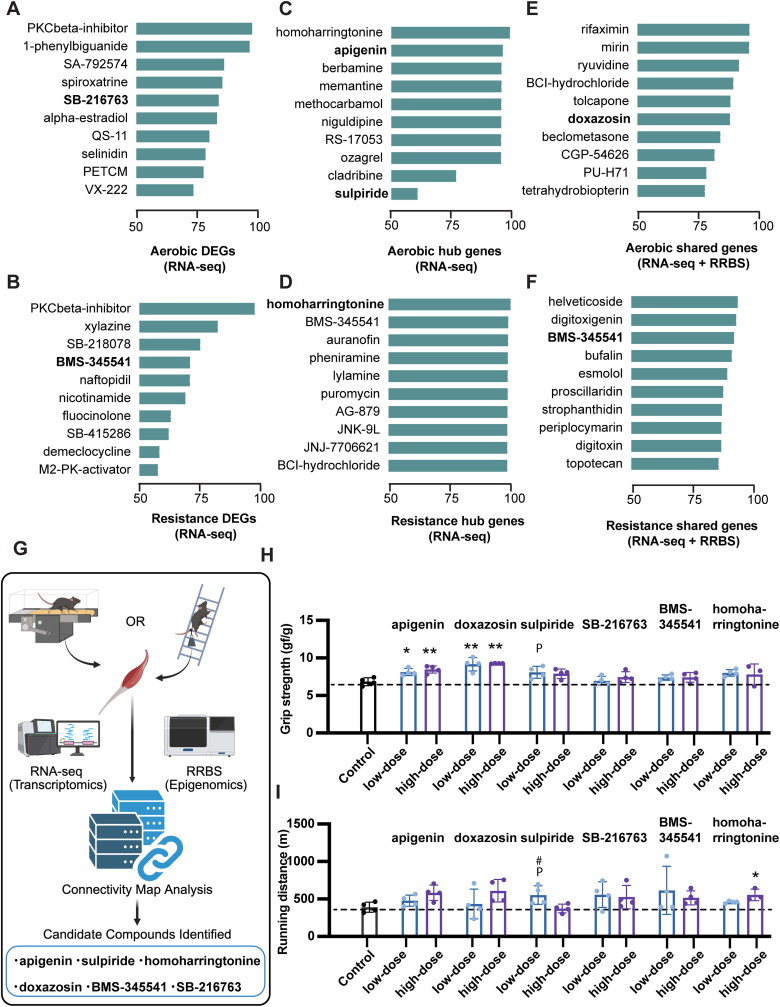
**Identification and in vivo validation of candidate exercise-mimetic compounds.** (A, B) Compounds with high connectivity scores based on differentially expressed genes following aerobic (A) or resistance (B) exercise. (C, D) The top 10 compounds identified based on highly connected genes (hub genes) derived from exercise-responsive PPI networks shown in Fig. 2G and H. (E, F) The top 10 compounds identified based on genes commonly altered in both RNA-seq and RRBS datasets after aerobic (E) or resistance (F) exercise. (G) Schematic illustration of the CMap analysis workflow. RNA-seq and RRBS analyses identified transcriptomic and epigenomic signatures following moderate aerobic or resistance exercise. These signatures were queried against the CMap database to identify candidate exercise-mimetic compounds with high connectivity scores. (H, I) In vivo validation of candidate compounds administered orally for 2 weeks in eight-week-old male C57BL/6J mice. (H) Grip strength. (I) Running distance to exhaustion on a treadmill. Dashed lines indicate the mean value of the vehicle-treated control group. Data are presented as mean ± SEM. Statistical significance was determined using one-way ANOVA followed by Tukey's post hoc test. ∗P < 0.05, ∗∗P < 0.01 vs. control group. P indicates a trend toward significance compared to control (P = 0.058 for sulpiride low-dose in grip strength test; P = 0.063 for sulpiride low-dose in treadmill running test). # indicates P < 0.05 between low- and high-dose groups (sulpiride).

Connectivity score analysis revealed several compounds with high similarity to the transcriptional signatures induced by moderate exercise (Fig. 4A–F). The compound selection workflow based on transcriptomic and/or epigenomic signatures is summarized in Fig. 4G. We identified apigenin, doxazosin, sulpiride, SB-216763, BMS-345541, and homoharringtonine as candidates for exercise-mimetic compounds with high connectivity scores. These compounds were chosen for their potential to emulate the diverse and beneficial effects of exercise. Notably, apigenin has been shown to ameliorate mitochondrial dysfunction [18], while doxazosin promotes the formation of C2C12 myoblasts, which is essential for muscle repair and regeneration [19]. Sulpiride is an FDA-approved antipsychotic, and SB-216763, a GSK-3β inhibitor, has been reported to mitigate skeletal muscle atrophy [20], reflecting its therapeutic potential. BMS-345541, a selective IκB kinase inhibitor, has shown efficacy against inflammation and joint destruction in arthritis models [21]. Homoharringtonine is FDA-approved for the treatment of chronic myeloid leukemia. To rapidly filter the candidate compounds identified via in silico CMap analysis, while adhering to the 3Rs (Replacement, Reduction, and Refinement) principle of animal welfare, we designed an initial, exploratory in vivo screen with a restricted sample size. Then, we examined the efficacy of these candidates in mimicking the effects of moderate exercise following the oral administration of each compound to healthy young mice for 2 weeks. Motor performance assessments revealed that apigenin and doxazosin significantly enhanced grip strength (Fig. 4H and I). Homoharringtonine significantly increased the treadmill running distance. Low-dose sulpiride showed a trend toward increased grip strength (P = 0.058) and running endurance (P = 0.063), while a significant difference was observed between low- and high-dose groups in running distance (P < 0.05), indicating possible inverse effects at higher doses. In contrast, SB-216763 and BMS-345541 did not produce notable effects on motor performance. No overt changes in organ weights were observed for apigenin and doxazosin during the 2-week administration, although homoharringtonine increased liver weight, raising concerns about hepatotoxicity (Fig. S4). These observations indicate that apigenin and doxazosin partially reproduce selected physiological outcomes of moderate exercise under short-term administration. The doses used are within ranges previously reported in rodent studies and below toxic thresholds [22,23], supporting short-term tolerability. Nevertheless, comprehensive evaluation of long-term safety and molecular mimicry will require more detailed toxicological studies.

### 2.6 Effects of apigenin, doxazosin, and sulpiride on skeletal muscle function and endurance in combination with exercise

**Fig. 5:**
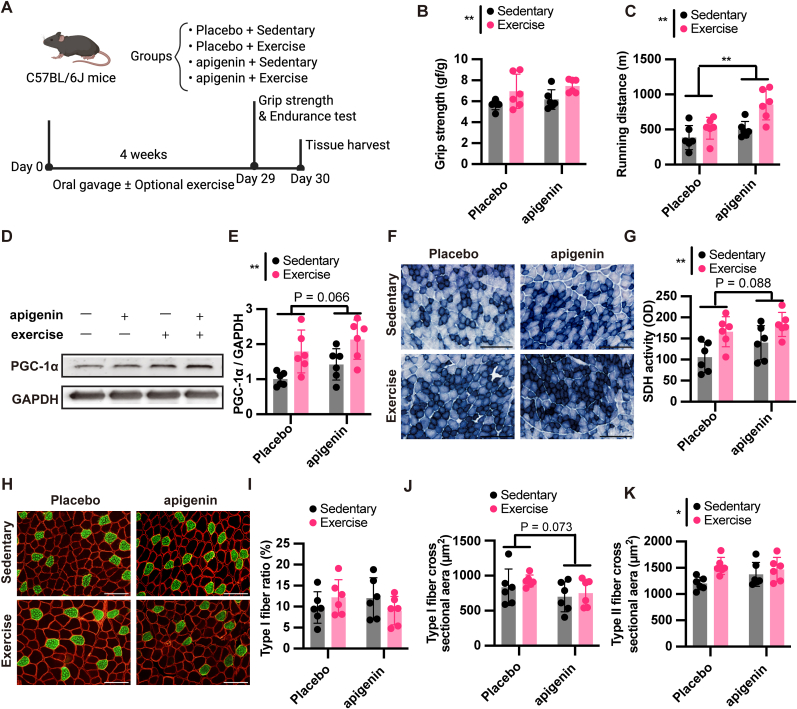
**Effects of apigenin and exercise on muscle performance and adaptation.** (A) Experimental design. Eight-week-old male C57BL/6J mice were randomly assigned into four groups: Placebo + Sedentary, Placebo + Exercise, apigenin + Sedentary, and apigenin + Exercise. Mice received daily oral gavage of apigenin (50 mg/kg/day) or vehicle for 4 weeks, with or without moderate exercise (treadmill at 20 m/min and ladder climbing with 120% body weight), based on prior optimization (see Fig. 1). (B, C) Motor performance outcomes following the 4-week intervention. (B) Grip strength and (C) treadmill running distance to exhaustion. (D, E) Protein levels of PGC-1α in the gastrocnemius muscle. (D) Representative Western blot images and (E) quantification of PGC-1α levels normalized to GAPDH. (F, G) SDH activity in the gastrocnemius muscle. (F) Representative images of SDH-stained sections and (G) corresponding quantification. Scale bars, 200 μm. (H–K) Immunohistochemical analysis of muscle fiber composition and size. (H) Representative images showing type I fibers (green, anti–MyHC I) and cell membranes (red, anti-dystrophin) in the gastrocnemius muscle. Scale bars, 100 μm. Quantitative analysis includes (I) type I fiber ratio, (J) type I fiber cross-sectional area, and (K) type II fiber cross-sectional area. Data are presented as mean ± SEM. Two-way ANOVA was used to evaluate the main effects of exercise and apigenin, as well as their interaction. Post hoc comparisons were performed using Šídák's multiple comparisons test. Statistical significance: ∗P < 0.05, ∗∗P < 0.01, ∗∗∗P < 0.001. Asterisks indicate a significant main effect of either exercise or apigenin administration. No significant interaction effects were observed for any parameter.

**Fig. 6:**
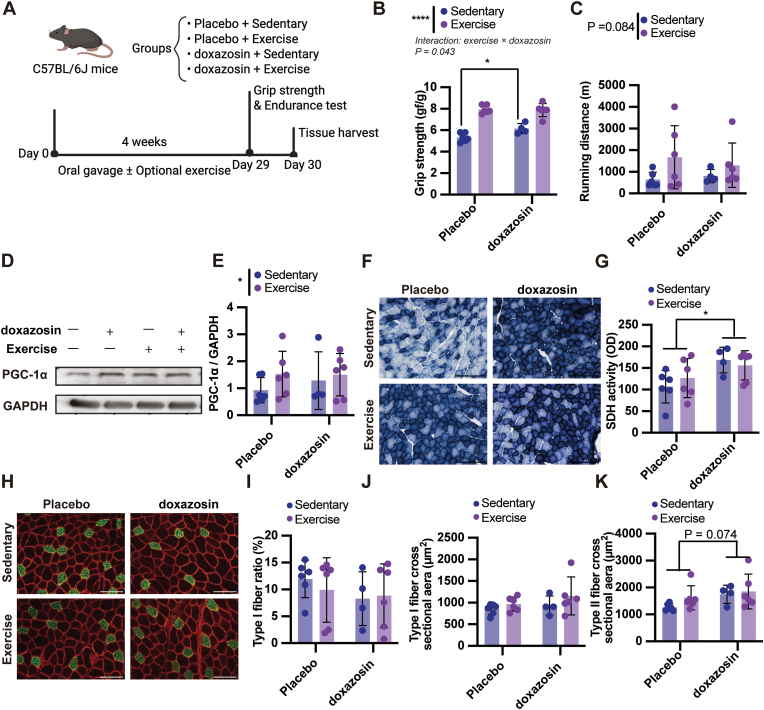
**Effects of doxazosin and exercise on muscle performance and adaptation.** (A) Experimental design. Eight-week-old male C57BL/6J mice were randomly assigned to four groups: Placebo + Sedentary, Placebo + Exercise, doxazosin + Sedentary, and doxazosin + Exercise. Mice received daily oral gavage of doxazosin (10 mg/kg/day) or vehicle for 4 weeks, with or without moderate exercise (treadmill and ladder climbing). Grip strength and endurance tests were performed on day 29, followed by tissue harvest on day 30. (B, C) Motor performance outcomes. (B) Grip strength and (C) treadmill running distance. For grip strength, a significant interaction between exercise and doxazosin was observed (P = 0.043). Post hoc comparisons revealed that doxazosin significantly increased grip strength in sedentary mice (P = 0.029). Furthermore, a highly significant main effect of exercise was observed across groups (P < 0.0001). (D, E) PGC-1α protein levels in the gastrocnemius muscle. (D) Representative Western blot images and (E) quantification normalized to GAPDH. (F, G) SDH activity in the gastrocnemius muscle. (F) Representative SDH-stained sections and (G) quantification. Scale bars, 200 μm. (H–K) Muscle fiber type and size analysis by immunohistochemistry. (H) Representative images of type I fibers (green, anti–slow MyHC) and cell membranes (red, anti-dystrophin). Scale bars, 100 μm. Quantification includes (I) type I fiber ratio, (J) type I fiber cross-sectional area, and (K) type II fiber cross-sectional area. Statistical analyses were performed using two-way ANOVA followed by Šídák's multiple comparisons test. For all panels except (B), only the main effects of exercise or doxazosin were observed and are reported accordingly. A significant interaction was detected only in (B), and post hoc comparisons for simple effects are shown. Data are presented as mean ± SEM. ∗P < 0.05, ∗∗P < 0.01, ∗∗∗P < 0.001, ∗∗∗∗P < 0.0001 for main effects, interactions, or post hoc group differences as specified.

To determine whether the effects of candidate exercise mimetics are enhanced when combined with physical activity, we examined the impact of apigenin, doxazosin, and sulpiride, with or without exercise, on skeletal muscle function and adaptation. Apigenin treatment for 4 weeks (Fig. 5A) had no effect on grip strength (Fig. 5B; drug effect: P = 0.236, power = 0.247; exercise effect: P = 0.006, power = 0.898), but significantly increased running endurance independent of exercise (Fig. 5C; drug effect: P = 0.004, power = 0.926; exercise effect: P = 0.002, power = 0.954). It also showed a trend toward increased PGC-1α protein levels (drug effect: P = 0.066, power = 0.524; exercise effect: P = 0.001, power = 0.978) and SDH activity (drug effect: P = 0.088, power = 0.462; exercise effect: P = 0.002, power = 0.962), with no interaction observed (Fig. 5D–G). Apigenin treatment did not alter the type I fiber ratio (Fig. 5I; drug effect: P = 0.791, power = 0.059; exercise effect: P = 0.867, power = 0.054) or type II fiber cross-sectional area (Fig. 5K; drug effect: P = 0.491, power = 0.113; exercise effect: P = 0.012, power = 0.816), whereas the type I fiber cross-sectional area showed a downward trend that did not reach statistical significance (Fig. 5J; drug effect: P = 0.073, power = 0.505; exercise effect: P = 0.389, power = 0.150). Next, we evaluated the effects of doxazosin (Fig. 6A). A significant interaction between doxazosin and exercise was detected for grip strength (Fig. 6B; interaction: P = 0.043, power = 0.621). Post-hoc analysis revealed that doxazosin significantly increased grip strength in sedentary mice (P = 0.029, power = 0.725), indicating that the effect of doxazosin may differ depending on exercise status. However, no significant difference was observed between the exercise alone and doxazosin + exercise groups, suggesting that the interaction reflects additive rather than synergistic effects. Endurance and PGC-1α protein levels were not affected (Fig. 6C–E; for endurance: drug effect P = 0.815, power = 0.057; exercise effect: P = 0.084, power = 0.478; for PGC-1α: drug effect P = 0.637, power = 0.079; exercise effect: P = 0.251, power = 0.236). SDH activity was significantly elevated by doxazosin independent of exercise (Fig. 6F and G; drug effect: P = 0.012, power = 0.825; exercise effect: P = 0.827, power = 0.056). A trend toward increased type II fiber cross-sectional area was observed in the doxazosin group (Fig. 6K; drug effect: P = 0.074, power = 0.509; exercise effect: P = 0.247, power = 0.239), while type I fiber ratio and cross-sectional area remained unchanged (Fig. 6H–J; for Type I ratio: drug effect: P = 0.309, power = 0.195; exercise effect: P = 0.745, power = 0.064; for Type I CSA: drug effect: P = 0.270, power = 0.190; exercise effect: P = 0.165, power = 0.279). Sulpiride treatment for 4 weeks showed no measurable effects on grip strength, endurance, PGC-1α protein, SDH activity, or fiber composition, consistent with the data presented in Fig. S5. Importantly, post-hoc power analyses suggested that the non-significant trends observed in both apigenin and doxazosin interventions represented large effect sizes. The lack of statistical significance in these markers may be attributed to potential Type II errors resulting from the modest sample size, whereas the primary functional outcomes (endurance for apigenin, strength for doxazosin) were adequately powered. Together, these results suggest mode-specific effects of apigenin and doxazosin on skeletal muscle, with apigenin enhancing endurance independent of exercise, and doxazosin improving strength with effects that may vary depending on exercise status.

To elucidate the specific molecular mechanisms underlying these divergent functional phenotypes, we evaluated key kinase pathways in skeletal muscle following chronic administration of the mimetics. Apigenin induced a broad-spectrum activation of critical energy-sensing and stress-adaptive modules, significantly upregulating the phosphorylation of AMPK, p38 MAPK, and ERK1/2 (Fig. S6). This comprehensive kinase activation aligns well with its consistent endurance-enhancing phenotype and muscle-preserving effects. Conversely, doxazosin elicited a highly specific upregulation of *p*-ERK1/2 without altering primary metabolic sensors like AMPK or p38 MAPK (Fig. S7). This highly targeted mechanotransduction-associated signaling matches its specific physiological profile of improving strength and musculoskeletal integrity without enhancing endurance capacity.

### 2.7 Therapeutic efficacy of exercise mimetics apigenin and doxazosin in disuse atrophy, osteoporosis, osteoarthritis, obesity, and aging

**Fig. 7:**
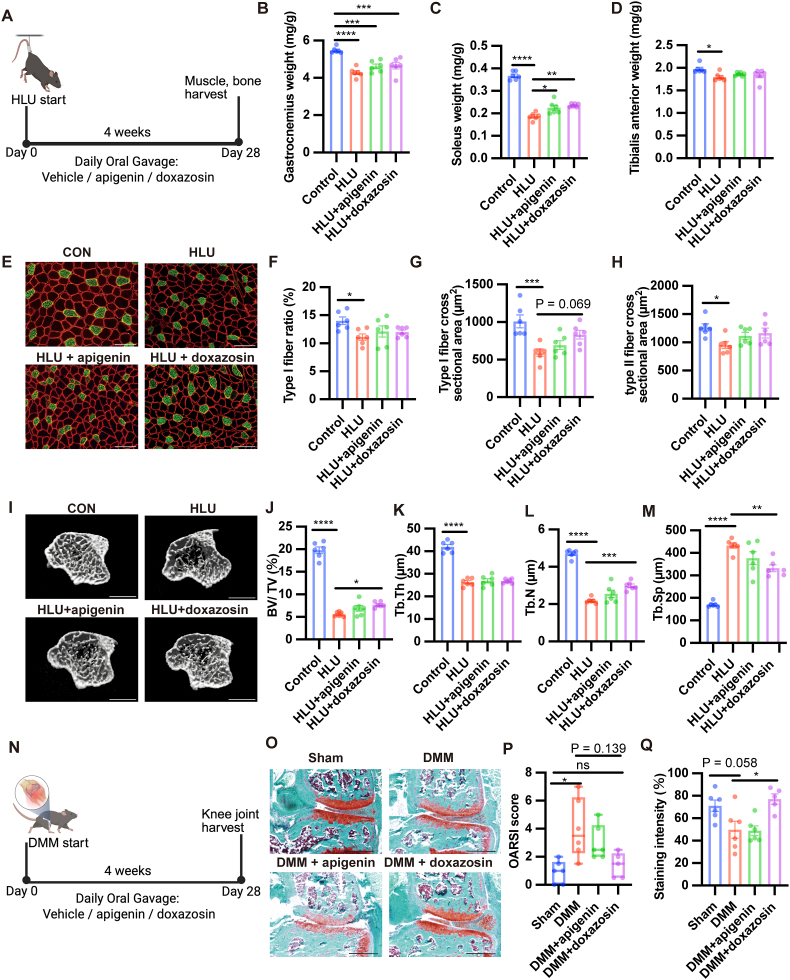
**Effects of apigenin and doxazosin on disuse-induced muscle and bone loss, and knee osteoarthritis.** (A–M) Hindlimb unloading (HLU) model. (A) Experimental timeline of HLU and daily oral administration of vehicle, apigenin (50 mg/kg/day), or doxazosin (10 mg/kg/day) for 4 weeks. (B–D) Skeletal muscle wet weights normalized to body weight: (B) gastrocnemius, (C) soleus, and (D) tibialis anterior. (E–H) Gastrocnemius muscle fiber typing analysis. (E) Representative immunofluorescence images. Type I fibers are stained green (anti–MyHC I), and cell membranes are labeled red (anti-dystrophin). Scale bars, 100 μm. Quantification of (F) type I fiber ratio, (G) type I fiber cross-sectional area, and (H) type II fiber cross-sectional area. (I–M) Micro–computed tomography (μCT) analysis of trabecular bone in the distal femur. (I) Representative μCT images. Scale bars, 1 mm. Quantitative parameters: (J) bone volume fraction (BV/TV), (K) trabecular thickness (Tb.Th), (L) trabecular number (Tb.N), and (M) trabecular separation (Tb.Sp). (N–Q) Destabilization of the medial meniscus (DMM)-induced osteoarthritis model. (N) Experimental timeline of DMM surgery and daily administration of compounds (vehicle, apigenin, or doxazosin) for 4 weeks. (O) Representative Safranin O/Fast Green–stained knee joint sections. Scale bars, 500 μm. Quantification of (P) modified OARSI histological scores and (Q) Safranin O-positive proteoglycan staining intensity. Data are presented as mean ± SEM, except for OARSI scores, which are shown as median with interquartile range. Statistical significance was determined using one-way ANOVA followed by Tukey's post hoc test. ∗P < 0.05, ∗∗P < 0.01, ∗∗∗P < 0.001, ∗∗∗∗P < 0.0001, ns: not significant.

The benefits of exercise extend beyond skeletal muscle, influencing bone, adipose tissue, and articular cartilage. We hypothesized that effective exercise-mimetic compounds would similarly exert therapeutic effects on multiple tissues. Therefore, we evaluated the therapeutic potential of apigenin and doxazosin in models of disuse-induced muscle and bone atrophy, osteoporosis, osteoarthritis, obesity, and aging. In a four-week HLU mouse model (Fig. 7A), treatment with either apigenin or doxazosin significantly preserved soleus muscle weight, which was otherwise reduced by disuse, while no significant changes were observed in the gastrocnemius or tibialis anterior muscles (Fig. 7B–D). Histological analysis showed no major differences in fiber-type distribution among groups (Fig. 7E and F). However, doxazosin tended to enlarge type I fibers without affecting type II fibers (Fig. 7G and H), which may reflect a partial preservation of slow-twitch muscle morphology under unloading conditions. Notably, doxazosin also attenuated HLU-induced bone loss. Micro-CT analysis revealed significant improvements in BV/TV, Tb.N, and Tb.Sp, whereas Tb.Th remained unchanged across groups (Fig. 7I–M).

We then assessed the efficacy of candidate exercise mimetics in traumatic knee osteoarthritis induced by DMM (Fig. 7N). OARSI scores were significantly higher in DMM mice, whereas scores in doxazosin-treated mice were not significantly different from those in sham mice (Fig. 7O and P). Additionally, we observed an increase in the intensity of safranin O staining, reflecting the higher proteoglycan content in the articular cartilage of doxazosin-treated DMM mice than in DMM mice (Fig. 7Q). These findings indicate that doxazosin protects articular cartilage integrity.

Next, we explored the potential therapeutic effects of our candidate exercise mimetics on bone loss due to EDOP. In contrast to the results obtained in the HLU model, we observed no therapeutic benefit of either apigenin or doxazosin in EDOP (Figure S8, A–E). These findings indicate that doxazosin effectively supports the maintenance of bone mass, primarily under conditions of limited loading, similar to those in the HLU model. Then, we examined the impact of apigenin and doxazosin on HFD–induced obesity and aging. Apigenin and doxazosin did not improve body weight, fat mass, or glucose tolerance (Figure S8, F–K). No beneficial effects were observed on aging-related parameters (Fig. S9).

In summary, both apigenin and doxazosin alleviated disuse-induced muscle atrophy. Doxazosin additionally demonstrated broader therapeutic efficacy by attenuating disuse-induced bone loss and traumatic osteoarthritis.

## 3 Discussion

This study aimed to elucidate the molecular mechanisms of moderate exercise-induced adaptations. Beyond skeletal muscle–intrinsic regulation, our integrative RNA-seq, RRBS, and phosphoproteomic analyses revealed significant enrichment of pathways related to the extracellular region, insulin/adipokine signaling, and FoxO signaling. These enrichments suggest that muscle-derived signals may contribute to cross-tissue communication and systemic adaptations to exercise. While experimental evaluation of non-musculoskeletal organs was beyond the scope of this study, the systemic context of these pathways highlights the broader relevance of our findings. As a secondary objective, we explored compounds that may mimic these effects. We report three main findings: (1) transcriptomic, epigenetic, and phosphoproteomic analyses revealed distinct and shared regulatory pathways in aerobic and resistance exercise, including MAPK, FoxO, and circadian signaling. (2) Apigenin and doxazosin partially mimicked exercise effects: apigenin improved endurance and mitochondrial markers, while doxazosin enhanced muscle strength and hypertrophy. (3) Apigenin exerted partial protective effects against disuse-induced muscle atrophy, whereas doxazosin provided broader therapeutic benefits in models of muscle atrophy, bone loss, and osteoarthritis.

Defining optimal exercise intensities is crucial for effectively maximizing physiological adaptations while minimizing adverse effects. Although exercise-induced adaptations are generally dose-dependent, excessively high intensities may trigger detrimental cellular stress responses, such as excessive oxidative stress, mitochondrial dysfunction, impaired protein homeostasis, and chronic inflammation [24,25]. Consistent with human studies [26], our data show that aerobic exercise at 20 m/min optimally enhances mitochondrial biogenesis—indicated by peak PGC-1α expression—without the oxidative stress observed at 24 m/min. Crucially, the stability of both lipid peroxidation markers (MDA) and primary antioxidant enzymes (Sod1) indicates that this molecularly defined moderate workload safely promotes adaptive remodeling with no overt changes in these selected oxidative stress markers. Likewise, resistance exercise at 120% of body weight maximizes muscle hypertrophy by balancing anabolic signaling and protein degradation, whereas intensities beyond this threshold fail to further enhance protein synthesis, likely due to excessive translational stress and defective protein accumulation [27]. These findings highlight the physiological relevance of moderate-intensity exercise and inform the design of safe and effective training protocols and mimetic interventions. Importantly, applying a dose–response framework allowed us to define these optimal intensities systematically, rather than relying on a single fixed protocol as in many prior rodent omics studies. By analyzing skeletal muscle responses at multiple intensities, we could identify the inflection point where adaptive signaling was maximized while excessive stress was avoided, thereby strengthening the mechanistic interpretation of our multi-omics data. Translationally, treadmill running at ∼20 m/min in mice corresponds to ∼70% $\dot{V}$ O_2_max, typically classified as moderate-vigorous exercise in humans [28]. Similarly, ladder climbing with ∼120% body weight maximally induces hypertrophic signaling in rodents, conceptually comparable to ∼70–80% of one-repetition maximum (1RM) in humans, the load recommended by the American College of Sports Medicine for hypertrophy training [29]. Although we observed inter-individual variability even among genetically similar mice, paralleling human heterogeneity, our use of cohort-level, not individualized, prescriptions means “optimal” here denotes a group-level optimum rather than per-mouse individualized intensity.

Our integrative transcriptomic, epigenetic, and phosphoproteomic analyses revealed coordinated regulatory programs that underlie the beneficial adaptations to moderate exercise. Across both aerobic and resistance modalities, we identified consistent modulation of key pathways—particularly insulin, AMPK, FoxO, and circadian rhythm—at multiple regulatory levels. At the transcriptional level, moderate exercise upregulated genes involved in mitochondrial biogenesis and protein folding (e.g., PGC-1α, Hspa1a), while suppressing components of proteasomal degradation and FoxO signaling (e.g., Ubb, Ubc), suggesting a shift toward an anabolic, stress-resilient state. Epigenetically, both modalities induced promoter demethylation in genes associated with metabolic regulation and vascular signaling (e.g., MAPK, AMPK, cGMP–PKG), with aerobic exercise showing broader demethylation and expression coupling than resistance exercise. Phosphoproteomic profiling further confirmed downstream activation of these pathways: aerobic exercise increased phosphorylation of proteins involved in glycolysis, cGMP–PKG, and MAPK signaling, while resistance exercise impacted insulin-related and contractile pathways. These findings collectively indicate that moderate exercise not only induces immediate transcriptional shifts but also reinforces longer-term gene accessibility and post-translational readiness, enabling sustained metabolic remodeling. These findings collectively suggest that moderate exercise orchestrates a multilayered regulatory network, with MAPK, FoxO, and circadian pathways serving as key convergence nodes for adaptation in skeletal muscle.

Through connectivity analysis of transcriptional signatures induced by moderate exercise, we initially identified candidate compounds associated with both aerobic and resistance exercise signatures. However, the compounds that demonstrated functional efficacy in vivo—apigenin and doxazosin—were ultimately linked to aerobic exercise-derived signatures. Apigenin, a naturally occurring flavonoid abundant in parsley, celery, and chamomile, possesses well-documented antioxidant properties with therapeutic potential against diabetes, hypertension, cancer, and neurodegenerative disorders [[30], [31], [32], [33], [34], [35]]. In our study, apigenin administration modestly increased endurance performance and exhibited a trend toward enhanced mitochondrial markers, including PGC-1α protein levels and SDH activity. Although these changes did not reach statistical significance, they are consistent with previous reports demonstrating apigenin's ability to mitigate oxidative stress and improve mitochondrial function in skeletal muscle [18,36], supporting its role as a candidate aerobic exercise mimetic. In the context of disuse-induced muscle atrophy, apigenin administration conferred partial protection, suggesting a role in preserving mitochondrial integrity and mitigating oxidative stress [37]. Mechanistically, apigenin has been reported to activate the AMPK–PGC-1α axis to promote mitochondrial biogenesis pathways [38,39] that likely underlie the endurance-enhancing effects observed. On the other hand, doxazosin, an FDA-approved antihypertensive α-1 adrenergic receptor antagonist [40], has been reported to promote muscle hypertrophy and strength enhancement. These effects suggest activation of the PI3K/Akt/mTOR pathway [41], which governs protein synthesis and muscle growth. Some previous studies indicate that doxazosin can activate the PI3K/Akt/mTOR pathway [41], which governs protein synthesis and hypertrophy, as well as promote myoblast fusion in C2C12 cells [19]. Its bone-protective effects may be mediated by ERK1/2 activation, which promotes osteoblast proliferation [42], while its chondroprotective actions likely reflect inhibition of α1-adrenergic signaling, which otherwise promotes chondrocyte apoptosis and matrix degradation [43]. Collectively, these effects are mechanistically consistent with activation of the PI3K-Akt-mTOR axis, ERK1/2-mediated osteoblast proliferation, and protection against chondrocyte apoptosis via α1-adrenergic blockade.

Exercise mimetics targeting specific molecular pathways, such as the AMPK activator AICAR and the PPARδ agonist GW501516, have previously demonstrated potent biological effects extending beyond typical physiological responses to exercise [44]. While this potent activation can yield impressive results, it also poses significant safety risks. For example, many AMPK activators directly inhibit mitochondrial ATP production, leading to adverse outcomes such as energy depletion, lactic acidosis, and potential tissue damage, particularly in high-energy-demand organs. [45]. In contrast, the effects elicited by apigenin and doxazosin in our study were comparatively modest relative to acute exercise. Our integrative analyses revealed that moderate exercise triggers coordinated responses involving multiple molecular pathways, resulting in cumulative and systemic physiological remodeling rather than abrupt or dramatic changes. Importantly, our use of CMap analysis enabled the unbiased identification of compounds that recapitulate this multidimensional transcriptional signature, thereby offering a mechanistically grounded approach to identifying candidate compounds that partially reproduce selected exercise-associated effects. Unlike previous candidate-based screens, CMap leverages global gene expression patterns to match exercise-induced molecular states with pharmacologically accessible profiles. Thus, the absence of dramatic effects seen with apigenin and doxazosin should be considered an advantage, reflecting their high fidelity in mimicking real exercise. Moderate exercise is defined by cumulative, adaptive responses rather than acute stimulation, and pharmacological agents that emulate this mode of action may offer safer, more physiologically relevant therapeutic strategies. Beyond our short-term study, prior investigations have explored the long-term effects of apigenin and doxazosin across multiple organ systems. Chronic apigenin supplementation has been shown to ameliorate dyslipidemia, hepatic steatosis, and insulin resistance [46,47], to improve cardiovascular health by restoring endothelial function and reducing vascular inflammation [48,49]. In addition, apigenin modulates immune responses by inhibiting NF-κB signaling and preventing systemic inflammatory damage [50]. For doxazosin, long-term α-blocker therapy has shown sustained efficacy in alleviating lower urinary tract symptoms in benign prostatic hyperplasia and chronic prostatitis, with improvements in both symptom scores and quality of life [51]. Together, these findings emphasize that future studies should extend beyond musculoskeletal outcomes to assess the safety and efficacy of apigenin and doxazosin across cardiovascular, immune, and nervous systems.

This study has some limitations. First, because our initial exercise intensity screening and multi-omics analyses were fundamentally based on a single bout of acute exercise, highly discriminative methods to evaluate immediate macroscopic functional changes were lacking. Consequently, we relied primarily on sensitive molecular markers at this early stage. Although these functional outcomes were explicitly validated in our subsequent downstream chronic models, the reliance on molecular markers during the initial screening phase remains a limitation. Related to these functional evaluations, the non-terminal assessments in our chronic models were evaluated post-intervention without baseline normalization. Future studies investigating exercise mimetics should employ repeated-measures (pre/post) designs to reduce inter-individual variability and improve the interpretability of modest functional effect sizes. Second, because the scope of our initial screening focused on identifying a safe intensity threshold using fundamental markers of lipid peroxidation (MDA) and primary antioxidant defense (Sod1), a comprehensive profiling of the skeletal muscle redox network (e.g., GSH/GSSG ratio, specific ROS handling markers) was not performed. Future studies should incorporate a broader redox panel to fully characterize the oxidative topology of moderate exercise. Third, the omics experiments had small group sizes. These assays were designed as exploratory screens, a strategy with precedent in prior studies [52,53]. Fourth, while we have identified apigenin and doxazosin as candidate exercise mimetics, the exact mechanisms underlying their effects were not elucidated. We hypothesized that apigenin may influence the signaling pathways related to oxidative phosphorylation capacity [37], and that doxazosin may affect pathways involved in protein synthesis [41]; however, further investigation is needed to confirm these mechanisms. Fifth, although this study defined moderate exercise in animals and identified potential exercise mimetics, we did not confirm their efficacy and safety in humans through clinical trials. To bridge this translational gap, we performed an in silico comparative analysis between our murine molecularly defined moderate-intensity transcriptomic signatures and a comprehensive human skeletal muscle exercise meta-analysis [54]. We identified an overlap of directionally conserved genes across species—including 50 concordant genes for acute aerobic exercise and 53 for acute resistance exercise (Fig. S10). This partial conservation of a subset of acute exercise-responsive pathways supports the clinical relevance of our murine model and provides potential translational relevance for the candidate mimetics derived from our pipeline. Additionally, apigenin, a naturally occurring flavonoid, is known for its low toxicity [22], and doxazosin, as an already approved drug, presents few safety concerns; this suggests that there are minimal barriers to the clinical application of these compounds as potential exercise mimetics. Finally, while the mimetics in this study showed benefits for skeletal muscle, bone, and articular cartilage, their effects on other systems altered by exercise, such as the brain, cardiovascular system, and immune system, have not been investigated and remain unknown. Nevertheless, as our candidate exercise mimetics effectively mimic post-exercise molecular events, it is plausible that they may also exert similar beneficial effects in these other tissues.

In conclusion, this study elucidated the molecular mechanisms underlying the beneficial adaptations to moderate-intensity aerobic and resistance exercise. Through integrated transcriptomic, epigenetic, and phosphoproteomic analyses, we characterized the upregulation of mitochondrial biogenesis, suppression of protein degradation pathways, and remodeling of circadian and insulin signaling as key regulatory features. As a secondary outcome, we identified apigenin and doxazosin as candidate exercise mimetics via transcriptome-based connectivity mapping. Both compounds, identified from aerobic exercise–derived signatures, demonstrated distinct but complementary effects. Apigenin partially recapitulated aerobic exercise by enhancing oxidative phosphorylation capacity and endurance, whereas doxazosin promoted muscle strength–related adaptations and provided protective effects on bone and articular cartilage. These findings suggest that pharmacological agents capable of eliciting partial, multi-layered responses akin to exercise may provide adjunct strategies to mitigate sedentary-lifestyle–related disorders.

## 4 Materials and methods

### 4.1 Animals

All experimental procedures were approved by the Institutional Animal Care and Use Committee (Approval No. P190814) and conducted in accordance with the Animal Experimentation Regulations of Kobe University. C57BL/6J mice (Japan SLC, Shizuoka, Japan) were used. Unless otherwise specified, male mice at 8 weeks of age were employed. Mice were housed in individually ventilated cages at 22 ± 1 °C, 55 ± 5% humidity, 12 h light/12 h dark, with ad libitum chow and water.

Disuse-induced muscle and bone loss was modeled by HLU as previously described [55]. Briefly, mice were anesthetized with 2% isoflurane and subcutaneously injected with buprenorphine (0.02 mg/kg) for analgesia. A sterile steel wire was inserted through an intervertebral space in the tail and shaped into a ring for suspension. The tail ring was connected to a ceiling-mounted track system, allowing free movement within the cage. The suspension angle was maintained at ∼30°, ensuring approximately 50% of body weight was borne by the forelimbs.

Knee osteoarthritis was induced by the DMM surgery under anesthesia as previously described [56]. Mice were anesthetized with 2% isoflurane and subcutaneously administered buprenorphine (0.02 mg/kg) for perioperative analgesia. The procedure involved incision of the medial capsule and transection of the anterior medial meniscotibial ligament on the right knee. Sham operations were performed in control animals using the same anesthetic and analgesic protocol. Detailed protocols for the ovariectomized model, high-fat diet, and aged models are provided in the Supplementary Materials.

### 4.2 Randomization, blinding, and inclusion/exclusion criteria

Animals were randomly assigned to the groups. Histological slides and muscle samples for omics, Western blotting, and biochemical assays were coded with randomly assigned identification numbers to blind investigators to group allocation. Functional tests were independently evaluated under blinded conditions. Omics analyses were processed under anonymized codes until completion of primary quality control. Prespecified exclusion criteria were: (i) death before the planned endpoint or meeting humane endpoints; (ii) major perioperative complications (e.g., failed recovery after DMM or HLU procedures); (iii) failure to complete exercise acclimation or to perform the prescribed training workload; and (iv) technical failure leading to unusable tissue.

### 4.3 Exercise protocol

For aerobic exercise, mice were acclimated for 5 days at 8 m/min (15 min on day 1, +15 min/day), followed by 2 rest days. The acute bout (n = 4 per group) was 60 min on a level treadmill (Muromachi Kikai Co., Ltd., Tokyo, Japan; MK-680) at fixed speeds of 10, 12, 14, 16, 18, 20, or 22 m/min (≈70–95% $\dot{V}$ O_2_max [28]). For chronic aerobic training, after acclimation, mice (n = 6–8 per group) performed once-daily sessions (7 days/week) for 60 min each, over 8 weeks, at fixed speeds of 12, 20, or 24 m/min. For resistance exercise, trained on a 110 cm ladder at 80° as described [57]. Acclimation occurred on 2 nonconsecutive days (4 sets × 3 climbs). The acute bout (n = 4 per group) consisted of 4 sets × 3 climbs performed at a fixed load assigned to each group (0%, 20%, 40%, 60%, 80%, 100%, 120%, 140%, or 160% of body mass). For chronic resistance training (n = 4 per group), following acclimation, sessions were conducted once daily for 8 weeks at fixed loads of 0%, 120%, or 160% of body mass. The maximal load of 160% body mass was selected based on pilot trials, in which mice were able to complete climbs up to this intensity but not at higher loads. Exercise sessions were conducted at fixed intensities, as our primary aim was to compare the effects of absolute workloads rather than progressive overload. Although relative workload may decrease with adaptation, the selected intensities remained within moderate-to-high ranges and were sufficient to elicit training-induced adaptations. For omics analyses (RNA-seq, RRBS, and phosphoproteomics), mice (n = 2 per group) underwent a single bout of moderate exercise (aerobic: 20 m/min treadmill; resistance: 120% body mass ladder climbing). All exercise sessions were performed during the light phase (10:00–15:00) under a standard 12 h light/12 h dark cycle; mice were not maintained on reversed cycles.

### 4.4 Sample collection

Gastrocnemius was analyzed because, in mice, it is a large, predominantly fast-twitch (type II-enriched) plantarflexor that is strongly recruited by both treadmill running and ladder-climbing tasks. Its mass provides sufficient yield for multi-omics assays across cohorts, enabling consistent sampling. While muscle-specific responses can differ (e.g., oxidative soleus vs. highly glycolytic EDL), the gastrocnemius offers a robust and widely used readout of exercise-induced adaptations. Mice were fasted for 12 h prior to tissue collection, with water available ad libitum. Sedentary controls underwent the same fasting and sampling schedule to control for circadian variation. A 12 h fasting period, though relatively long for mice, is widely employed in metabolic and exercise physiology studies to minimize postprandial variation [58,59]. For tissue collection, gastrocnemius muscles were harvested 2 h after acute aerobic exercise and 6 h after acute resistance exercise to capture early (aerobic) versus delayed (resistance) transcriptional waves, while multi-omics samples were collected immediately post-exercise to capture transient kinase signaling. For chronic interventions, samples were collected 48 h (aerobic) or 72 h (resistance) after the final session to minimize acute carryover and reflect training adaptations. We selected 6 h after acute resistance exercise because transcriptional programs downstream of mechanical/contractile signaling peak later than immediate early genes [60], whereas aerobic exercise elicits earlier transcriptional changes [61].

### 4.5 Administration of compounds

Mice (n = 3–4 per group) received daily oral gavage of six candidate compounds at low and high doses: apigenin (10 or 50 mg/kg/day; HY-N1201, MedChemExpress, Monmouth Junction, NJ, USA), doxazosin (1 or 10 mg/kg/day; S5782, Selleck Chemicals, Houston, TX, USA), sulpiride (10 or 50 mg/kg/day; HY-B1019, MedChemExpress), homoharringtonine (0.05 or 2.5 mg/kg/day; HY-14944, MedChemExpress); BMS-345541 (10 or 25 mg/kg/day, HY-10519, MedChemexpress) and SB-216763 (10 or 50 mg/kg/day; HY-12012, MedChemexpress). Dimethyl sulfoxide (DMSO), 5% (v/v) in 0.01 M phosphate-buffered saline (PBS), was used as the solvent for all compounds. Control animals received vehicle only. For 4-week follow-up studies, mice were treated once daily with apigenin (50 mg/kg/day; n = 6 per group), doxazosin (10 mg/kg/day; n = 4–6 per group), or sulpiride (20 mg/kg/day; n = 6 per group), either alone or in combination with moderate exercise, defined as a combination of treadmill running (20 m/min) and ladder climbing (120% body mass). For disease models, apigenin (50 mg/kg/day) and doxazosin (10 mg/kg/day) were administered daily for 4 weeks: 1) HLU: 8-week-old males (n = 6 per group). 2) DMM: 8-week-old males (n = 5–6 per group). 3) EDOP: 8-week-old females, n = 6 per group. 4) HFD: 4-week-old males, fed HFD for 8 weeks before 4-week compound treatment (n = 5–6 per group). 5) Aged cohort: 78-week-old males (n = 4–6 per group).

### 4.6 Grip strength test

Grip strength was assessed 24 h after the final session of exercise or compound administration using a digital force gauge (IMADA, Aichi, Japan). During each trial, mice were placed on the device and gently pulled backward until they released their grip. Each mouse underwent five trials, with 1 min of rest between each trial. The average of the three middle values—excluding the highest and lowest measurements—was used as the representative grip strength for each mouse.

### 4.7 Endurance running capacity test

An exhaustion test was conducted 24 h after the final session of exercise or compound administration to evaluate endurance running capacity, using an electric shock grid to enhance motivation. The mice were acclimated to the treadmill by running at 10 m/min at a grade 0% for 10 min on the day before the test. During the test, the treadmill was started at an initial speed of 10 m/min and a grade of 0%. The speed was increased by 2 m/min every 2 min up to a maximum of 30 m/min until the mice reached exhaustion, which was defined as a mouse remaining in continuous contact with the shock grid for 5 s. Endurance capacity was measured by the total running distance in meters.

### 4.8 SUnSET and western blotting

To evaluate muscle protein synthesis, the SUnSET method was employed as previously described [62]. To minimize dietary influences, mice were fasted for 12 h prior to tissue collection. Under deep anesthesia induced by 2% inhaled isoflurane, puromycin (0.04 μmol/g body weight; diluted in phosphate-buffered saline) was intraperitoneally injected. Thirty minutes after puromycin administration, the gastrocnemius muscles were collected and snap-frozen. Frozen gastrocnemius muscles were homogenized using a Bio Masher II (FUJIFILM Wako, Osaka, Japan) in extraction buffer containing 20 mM Tris-HCl (pH 7.5), 150 mM NaCl, 1% Triton X-100, 1 mM EDTA, 5 mM EGTA, a protease inhibitor cocktail (PIC-2, ITSI-Biosciences, Johnstown, PA, USA), and a phosphatase inhibitor cocktail (#78440, Thermo Fisher Scientific). For the SUnSET assay, homogenates were centrifuged at 500×*g* for 5 min at 4 °C, whereas for standard Western blotting, centrifugation was performed at 12,000×*g* for 15 min at 4 °C. The resulting supernatants were used for protein analysis. Protein concentrations were determined using the BCA Protein Assay Kit (TaKaRa, Shiga, Japan). All protein samples were adjusted to equal concentrations and mixed with 2 × Laemmli sample buffer. Proteins were separated on Mini-PROTEAN TGX precast gels (Bio-Rad Laboratories, Hercules, CA, USA; Trans-Blot Turbo system) and transferred to membranes using the Trans-Blot Turbo system. For the SUnSET assay, membranes were incubated with an anti-puromycin antibody (clone 12D10, 1:1000; Merck Millipore, Darmstadt, Germany) using the iBind Western System (Thermo Fisher Scientific, Waltham, MA, USA) along with iBind solution and an anti-mouse IgG-Fc2a secondary antibody (1:10000; Jackson Immuno Research, West Grove, PA, USA) for 2.5 h at room temperature. For standard Western blotting, membranes were blocked in 5% skim milk in TBS-T (Tris-buffered saline with 0.1% Tween-20) for 1 h at room temperature and incubated overnight at 4 °C with primary antibodies against peroxisome proliferator-activated receptor gamma coactivator 1-alpha (PGC-1α) (#ab54481, 1:3000; Abcam, Cambridge, UK), phospho-AMPKα (Thr172) (#2535, 1:1000; Cell Signaling Technology, Danvers, MA, USA), phospho-p38 MAPK (Thr180/Tyr182) (#9211, 1:1000; Cell Signaling Technology), phospho-p70 S6 Kinase (Thr389) (#9205, 1:1000; Cell Signaling Technology), phospho-p44/42 MAPK (ERK1/2) (Thr202/Tyr204) (#9101, 1:1000; Cell Signaling Technology), and GAPDH (#2118, 1:10000; Cell Signaling Technology, Danvers, MA, USA). Detection was performed using secondary antibodies (#A16110, Thermo Fisher Scientific). All immunoreactive bands were visualized using ImmunoStar LD (FUJIFILM Wako), and images were captured with the OptimaShot CL-420α system. Band intensities were quantified using ImageJ (NIH, Bethesda, MD, USA). For the SUnSET assay, puromycin signal intensities were normalized to total protein levels determined by Coomassie Brilliant Blue (CBB) R-350 staining (Bio-Rad). For standard Western blotting, PGC-1α protein levels were normalized to GAPDH as a loading control.

### 4.9 Malondialdehyde (MDA) analysis

Lipid peroxidation in the gastrocnemius muscle was assessed using a TBARS assay kit (Cayman Chemical, MI, USA), which quantifies MDA levels. Muscle homogenates were reacted with thiobarbituric acid reagents according to the manufacturer's instructions, and absorbance was measured at 540 nm.

### 4.10 Mitochondrial DNA (mtDNA) quantification

Total DNA was extracted from frozen gastrocnemius muscle using the DNeasy Blood & Tissue Kit (Qiagen, Hilden, Germany) according to the manufacturer's instructions. The concentration and purity of the extracted DNA were assessed by measuring the OD 260/280 ratio using a BioPhotometer D30 (Eppendorf, Hamburg, Germany). The relative mtDNA content was determined by quantitative PCR using primers specific for mitochondrial-encoded NADH dehydrogenase subunit 1 (ND1; Mm04225274_s1) and normalized to ANG (Mm00833184_s1). The relative abundance of mtDNA was calculated using the ΔΔCt method and expressed as fold-change relative to the control group.

### 4.11 RNA extraction and real-time PCR

The gastrocnemius muscle was completely homogenized in QIAzol Lysis Reagent (Qiagen). Total RNA was extracted using the Rneasy Plus Universal Mini Kit (Qiagen) in accordance with the manufacturer's protocols. The quality of the isolated RNA was investigated by measuring the OD 260/280 ratio with BioPhotometer D30 (Eppendorf). Reverse transcription was conducted using total RNA and the TaqMan Fast Virus 1-Step Master Mix (Thermo Fisher Scientific). mRNA expression was analyzed by quantitative real-time PCR using the StepOne real-time PCR system (Applied Biosystems, Foster City, CA, USA) and the Taqman Gene Expression Assay (Applied Biosystems). The relative mRNA expression was measured by the ΔΔCt method, with GAPDH (Mm99999915_g1) used as the control gene. The target gene information was as follows: PGC-1α (Ppargc1a: Mm01208835_m1), MuRF-1 (Muscle RING-finger protein-1; Mm01185221_m1), and Atrogin-1 (also known as MAFbx; Mm04207378_g1), Sod1 (Mm01344233_g1), Per2 (Mm00478099_m1), Bmal1 (Arntl: Mm00500223_m1), and Rev-erbα (Nr1d1: Mm00520708_m1).

### 4.12 RNA sequencing analysis

Total RNA was extracted from skeletal muscle tissue, and libraries were prepared using the NEBNext Ultra Directional RNA Library Prep Kit for Illumina (New England Biolabs, Ipswich, MA, USA), following the manufacturer's protocol. RNA was extracted from two biologically independent samples per group. This exploratory sample size was constrained by tissue availability and resources. Quality control was stringently applied at all steps (RNA integrity, library depth, mapping), and differential expression was analyzed using DESeq2 with variance shrinkage to stabilize estimates under small-n conditions. Accordingly, the RNA-seq results are presented as hypothesis-generating. The resulting libraries contained fragments ranging from 220 to 700 base pairs in length. After adapter ligation and PCR amplification, the libraries were sequenced on an Illumina NovaSeq 6000 platform (Illumina, San Diego, CA, USA), generating 150-base paired-end reads. Raw reads were trimmed using Trimmomatic to remove low-quality bases and adapter sequences. Quality control was performed both before and after trimming using FastQC (v0.11.4). On average, 50 million reads were obtained per sample. Filtered reads were then aligned to the reference transcriptome using HISAT2. Transcript abundance was quantified with featureCounts, and genes with low average read counts across samples were excluded from downstream analysis. Differential gene expression analysis was performed using the DESeq2 package, with significance defined as a false discovery rate (FDR)–adjusted p < 0.05 and an absolute log_2_ fold change (|log_2_FC|) ≥ 0.585 (≥1.5-fold change).

### 4.13 Reduced representation bisulfite sequencing (RRBS)

RRBS was performed to analyze DNA methylation patterns. Genomic DNA (1 μg per sample) from gastrocnemius muscle was digested with *Msp*I (New England Biolabs, Ipswich, MA, USA) and processed using the Zymo-Seq RRBS Library Kit (Zymo Research), followed by end repair, 3′-adenylation, and adapter ligation using the TruSeq ChIP-Seq Sample Preparation Kit (Illumina). Libraries were pooled and sequenced on an Illumina NovaSeq 6000 platform (paired-end, 2 × 150 bp). Only autosomal CpG sites with a sequencing depth ≥10, common to all samples, were used for downstream analyses. CpG sites were annotated by genomic and regional categories and assigned to the nearest gene within 1 Mbp. Genes were considered differentially methylated if they contained promoter-region CpG sites (TSS −1000 bp to 0 bp) showing significant methylation changes (FDR <0.05).

### 4.14 Phosphoproteome analysis

Quantitative phosphoproteomics was performed using IBT (Isobaric Tagging Technology)–based labeling and TiO_2_ enrichment. Gastrocnemius muscles from six mice (two each for control, aerobic, and resistance groups) were lysed, digested with trypsin, labeled with IBT reagents, and enriched for phosphopeptides using TiO_2_ affinity chromatography. Peptides were fractionated and analyzed by LC-MS/MS on a Q-Exactive HF-X mass spectrometer (Thermo Fisher Scientific). MS data were processed with Proteome Discoverer v1.4 using the MASCOT 2.3 search engine against the *Mus musculus* UniProt database (17,014 sequences). Carbamidomethylation (C) and IBT labeling (N-term, K) were set as fixed modifications; oxidation (M), deamidation (NQ), acetylation (N-term), and phosphorylation (STY) as variable modifications. FDR was set to 1% for peptide-spectrum matches. Phosphorylation site localization was evaluated using phosphoRS with a threshold of ≥0.75. In total, 1794 phosphorylation sites were quantified across 647 phosphoproteins, of which 203 (aerobic vs. control), 137 (resistance vs. control), and 7 (aerobic vs. resistance) met the criteria. Differential phosphopeptides were defined as those with fold change >1.2 or <0.833 and nominal p < 0.05. Given the limited sample size, no site-level multiple-testing correction was applied; this analysis should therefore be considered exploratory and hypothesis-generating. Hierarchical clustering, GO/KEGG enrichment, subcellular localization, and motif analysis were conducted on the differentially phosphorylated proteins. All analyses were performed by BGI (Shenzhen, China), and raw data are available upon request.

### 4.15 KEGG pathway enrichment analysis

KEGG pathway enrichment analysis was performed across transcriptomic (RNA-seq), epigenomic (RRBS), and phosphoproteomic datasets to elucidate the biological functions associated with exercise-induced molecular changes. DEGs were defined as those with adjusted p-values (padj) < 0.05, and were identified separately for aerobic and resistance exercise groups. DMGs were defined as genes that harbored significantly differentially methylated CpG sites (FDR <0.05) in their promoter regions (TSS −1000 bp to 0 bp), as determined by RRBS analysis. Differentially phosphorylated proteins were determined by fold change thresholds >1.2 or <0.833 with p < 0.05 from phosphoproteomic comparisons. To identify modality-specific and shared gene responses, DEGs from aerobic and resistance exercise conditions were compared for overlaps. Functional enrichment analysis was conducted as an overrepresentation analysis using the DAVID Bioinformatics Resources (v6.8; https://david.ncifcrf.gov/), with the *Mus musculus* genome as background. Differentially expressed or modified gene/protein lists were compared to the background, and pathways with enrichment p < 0.05 were considered significant. The resulting enriched pathways were visualized using SRplot (https://www.bioinformatics.com.cn/en).

### 4.16 Protein–protein interaction (PPI) network construction and hub gene identification

To investigate the functional interactions among DEGs identified from RNA-seq analysis, a PPI network was constructed using the STRING database (v11.5; https://string-db.org/) with a minimum required interaction score of >0.9. Disconnected nodes were hidden from the network. The interaction network was visualized in Cytoscape software (v3.8.2), and hub genes were identified using the cytoHubba plugin (v0.1) based on a comprehensive ranking that integrated six topological algorithms (MCC, MNC, Degree, EPC, Closeness, and Radiality). The top 10 genes with the highest combined scores were defined as hub genes.

### 4.17 CMap analysis

To identify candidate compounds that mimic the molecular effects of moderate exercise, we performed transcriptome-based drug repurposing using the next-generation CMap analysis platform (https://clue.io) developed by the Broad Institute [63]. Three gene sets were independently constructed based on RNA-seq and RRBS results: (1) all DEGs identified by RNA-seq; (2) hub genes among RNA-seq DEGs, identified via protein–protein interaction analysis; (3) genes commonly altered in both RNA-seq and RRBS analyses. Each gene set was divided into upregulated and downregulated genes and submitted to the CMap “Touchstone” query tool. The platform compared these exercise-induced gene signatures to a reference database of compound-treated gene expression profiles and returned ranked lists of small molecules with associated connectivity scores (range: −100 to +100). Compounds with high connectivity scores were considered strong exercise-mimetic candidates, as they were predicted to induce transcriptomic changes highly similar to those of moderate exercise.

### 4.18 Histological analyses

Succinate dehydrogenase (SDH) activity was assessed in frozen sections (10 μm) of the gastrocnemius muscle as previously described [64]. Sections were incubated in sodium phosphate buffer (pH 7.4) containing sodium succinate and tetranitro blue tetrazolium at 37 °C for 30 min in the dark. The reaction was stopped with 0.01 N HCl, and slides were rinsed and mounted. Hematoxylin and eosin staining was used to evaluate general muscle morphology. For cartilage evaluation, undecalcified frozen sections of the knee joint were prepared according to the method described by Kawamoto [65]. Sections were stained with safranin O and fast green. Cartilage degeneration was scored according to the Osteoarthritis Research Society International (OARSI) guidelines [66] by three blinded observers. All histological images were acquired using an Olympus BX53 microscope, and fiber cross-sectional area, SDH activity (optical density), and safranin O staining intensity were quantified using ImageJ (NIH) [67].

### 4.19 Immunofluorescence

Frozen gastrocnemius muscle sections (10 μm) were air-dried, fixed in 4% paraformaldehyde, and blocked with mouse IgG blocking reagent (MKB-2213a, Vector Laboratories) and 10% normal goat serum (VEC S-1000, Vector Laboratories) for 1 h at room temperature. Sections were incubated with the anti-slow myosin heavy chain (#ab11083, Abcam) at a 1:1000 dilution and anti-dystrophin antibody (#ab15277, Abcam) at a 1:1000 dilution. Secondary antibodies included Alexa Fluor 555-conjugated anti-rabbit IgG and Alexa Fluor 488-conjugated anti-mouse IgG1 (Thermo Fisher Scientific). Sections were mounted using ProLong Diamond Antifade Mountant. ImageJ (NIH) was used for semi-automated analysis of immunofluorescence images, with more than 300 muscle fibers analyzed per sample. Images were captured at 20 × magnification. The cross-sectional area and relative proportion of type I and type II fibers were quantified.

### 4.20 Micro-computed tomography (μCT) analysis

Using a micro-3D X-ray CT system (R_mCT2; Rigaku, Tokyo, Japan) at an isotropic voxel size of 20 μm (90 kV, 160 μA, 3 min per sample). Three-dimensional reconstruction and quantitative analysis were performed using TRI/3D-BON software (Ratoc, Tokyo, Japan). A 2 mm ROI was selected from the metaphysis below the growth plate. Trabecular and cortical bone were segmented using density thresholds of 650 and 750 mg HA/cm^3^, respectively. Parameters including bone volume fraction (BV/TV), trabecular thickness (Tb.Th), trabecular number (Tb.N), and trabecular separation (Tb.Sp) were evaluated.

### 4.21 Statistical analysis

Statistical analyses were performed by EZR (Saitama Medical Centre, Jichi Medical University, Saitama, Japan), which is a graphical user interface for R (The R Foundation for Statistical Computing, Vienna, Austria) [68]. The normality of each dataset was assessed using the Shapiro-Wilk test. Outliers were evaluated according to pre-specified criteria, and no data points were excluded. For comparisons among multiple groups, parametric data were analyzed using one-way analysis of variance (ANOVA) followed by Tukey's post hoc test. Nonparametric data (e.g., OARSI scores) were analyzed using the Kruskal-Wallis test with Steel-Dwass post hoc comparisons. In experiments involving two independent variables— exercise mimetic administration (apigenin, doxazosin, or sulpiride) and moderate exercise intervention—a two-way ANOVA was conducted to assess main effects and interaction effects. When significant interaction effects were detected, Šídák's multiple comparisons test was performed to compare individual group means. Furthermore, to address the statistical detection limits for the 4-week interventions, post-hoc power analyses (1 - β) were conducted for key endpoints based on the effect sizes (e.g., Cohen's *f*) derived from the variance explained by the main and interaction effects. Results are presented as mean ± standard error of the mean (SEM) for parametric data and as interquartile range for nonparametric data. Statistical significance was defined as p < 0.05.

## CRediT authorship contribution statement

**Hanlin Jiang:** Data curation, Investigation, Methodology, Validation, Visualization, Writing – original draft, Writing – review & editing. **Shota Inoue:** Investigation, Methodology, Visualization, Writing – original draft, Writing – review & editing. **Junpei Hatakeyama:** Investigation, Writing – review & editing. **Hideki Moriyama:** Conceptualization, Funding acquisition, Methodology, Supervision, Writing – original draft, Writing – review & editing.

## Declaration of competing interest

The authors declare that they have no known competing financial interests or personal relationships that could have appeared to influence the work reported in this paper.

## Data Availability

Data will be made available on request.
