# Supplementary material for: Multi-omics reveals molecular signatures of moderate intensity exercise and identifies candidate exercise mimetics in mice
Source: Redox Biol. 2026 Apr 22;93:104186. doi: 10.1016/j.redox.2026.104186 (PMC13112403; doi:10.1016/j.redox.2026.104186)
Supplement: Multimedia component 1 [file mmc1.docx]

***Multi-Omics Reveals Molecular Signatures of Moderate Intensity Exercise and Identifies Candidate Exercise Mimetics in Mice***

Hanlin Jiang,^1^ Shota Inoue,^1^ Junpei Hatakeyama,^1^ Hideki Moriyama^2*^

*Corresponding author: morihide@harbor.kobe-u.ac.jp

**Supplemented Methods**

**In Silico Cross-Species Transcriptomic Comparison**

To evaluate the translational relevance of the molecular signatures identified in our murine moderate-intensity exercise model, we performed a comparative analysis using a comprehensive human skeletal muscle exercise meta-analysis dataset (Pillon et al., 2020). Murine differentially expressed genes (DEGs) identified from our RNA-seq analysis were converted to their human orthologs using the g:Orth tool (g:Profiler). We then intersected these orthologs with human DEGs derived from acute aerobic and acute resistance exercise datasets. Directional consistency was defined as genes exhibiting the same direction of change (co-upregulated or co-downregulated) with an adjusted P-value < 0.05 in both the murine and human datasets.

**Ovariectomy (EDOP model)**

Eight-week-old female mice were subjected to bilateral ovariectomy to induce postmenopausal osteoporosis. Briefly, the mice were anesthetized with isoflurane inhalation. Incisions were made in the dorsal skin, muscles, and peritoneum, and the ovaries were excised along with the peri-ovarian fat. The success of the ovariectomy procedure was verified by measuring the uterine wet weight at the end of the experimental period. A sham operation was conducted using the same procedure without ovariectomy. For μCT analysis, the distal femurs were harvested to assess trabecular and cortical bone microarchitecture.

**HFD-induced obesity in mice**

After 1 week of acclimation to a normal diet, four-week-old male mice were randomly divided into four groups (HFD only, HFD + apigenin, HFD + doxazosin, and control without intervention) and provided with the appropriate diet. The normal diet used was CE-2, containing 4.8 wt% fat, 25.1 wt% protein, and 4.2 wt% carbohydrate, and the HFD was HFD-32, containing 31.9 wt% fat, 24.5 wt% protein, and 7.1 wt% carbohydrate; both diets were obtained from CLEA Japan Inc. (Tokyo, Japan). To assess glucose metabolism, an intraperitoneal glucose tolerance test was performed after 12 weeks of dietary intervention. Following a 6-hour fast, each mouse received an intraperitoneal injection of D-glucose (2.0 g/kg body weight). Blood glucose levels were measured at 0 (baseline), 15, 30, 60, and 120 minutes post-injection using a glucose meter (Accu-Chek Guide; Roche DC Japan Co., Ltd., Tokyo, Japan). Blood was collected from the tail vein, and the area under the curve (AUC) of glucose concentrations was calculated to evaluate glucose tolerance.

**Aged mice**

Seventy-eight-week-old male mice were used to assess the effect of exercise mimetics on aging. The mouse body weight was recorded, and the effects of aging were determined by measuring body weight, grip strength, and performing the four-limb wire-hanging test and rotarod test. There was a period of at least 1 hour between each test.

**Wire-hanging test**

To assess the muscular strength and endurance of the forelimbs and hindlimbs, we performed a four-limb wire-hanging test. Mice were placed on a wire grid (20 cm square with 1 cm × 1 cm spacing) elevated 45 cm above the ground. After an initial period of 5 seconds, the grid was inverted to start the test. The wire hanging time was recorded as the time until the mouse fell, up to a maximum of 60 seconds. Each mouse underwent three trials, and the average time to fall was calculated. To minimize the effects of fatigue, a rest period of 10 minutes between trials was provided.

**Rotarod test**

To evaluate motor coordination in aged mice, the accelerated rotarod test was utilized. To acclimate, mice were placed on the resting drum of a rotarod apparatus (MK-670, Muromachi Kikai, Tokyo, Japan) for at least 1 minute. The rotarod speed was then increased from 4 to 40 revolutions per minute (rpm) over 300 seconds. We conducted three trials per mouse, with a 15-minute interval between each trial to prevent fatigue. The duration for which each mouse remained on the rotating drum was recorded for each trial, and the average retention time was calculated.

**Supplemented Figures**

**
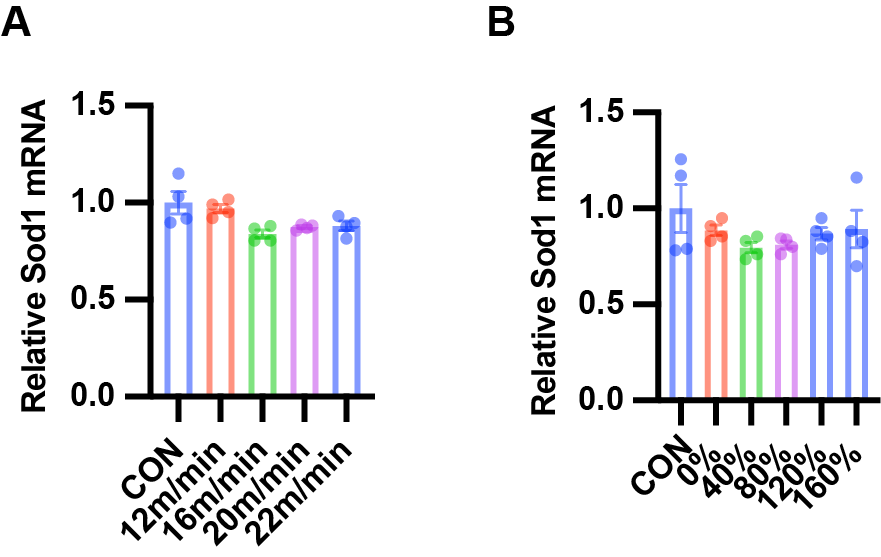
**

**Figure S1. Transcriptional evaluation of redox homeostasis across diverse exercise modalities.**

Relative mRNA expression levels of Sod1 following acute aerobic exercise (A) and acute resistance exercise (B) remained entirely stable across all tested intensities. Data are presented as mean ± SEM (n = 4 per group). Statistical significance was determined by one-way ANOVA.


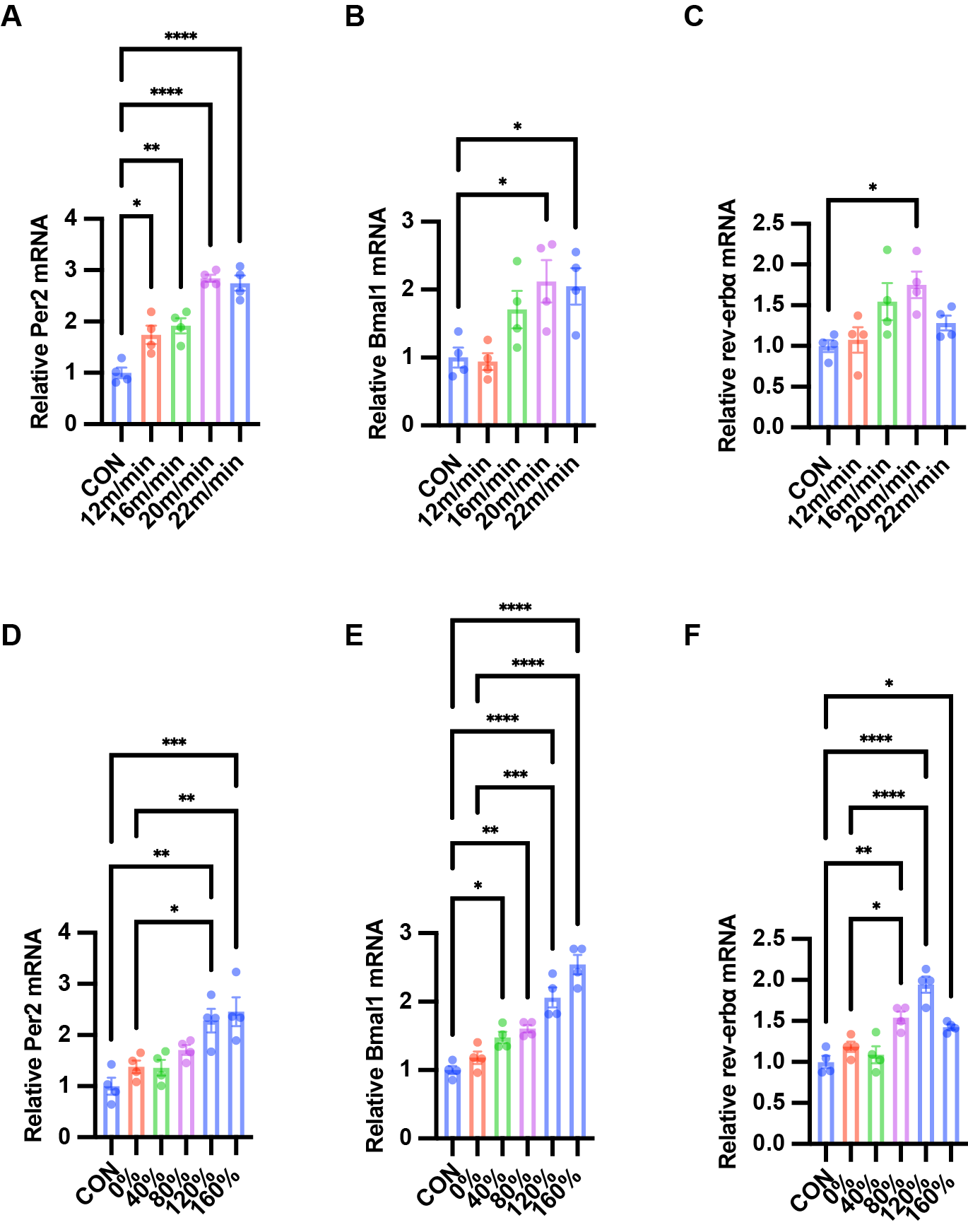


**Figure S2. Transcriptional validation of core circadian rhythm genes following acute exercise.**

(A–C) Relative mRNA expression levels of Per2 (A), Bmal1 (B), and rev-erbα (C) in skeletal muscle after acute aerobic exercise at varying intensities. (D–F) Relative mRNA expression levels of Per2 (D), Bmal1 (E), and rev-erbα (F) in skeletal muscle after acute resistance exercise at varying intensities. Data are presented as mean ± SEM (n = 4 per group). Statistical significance was determined using one-way ANOVA followed by Tukey’s post-hoc test. *P < 0.05, **P < 0.01, ***P < 0.001, ****P < 0.0001 for the indicated comparisons.

**
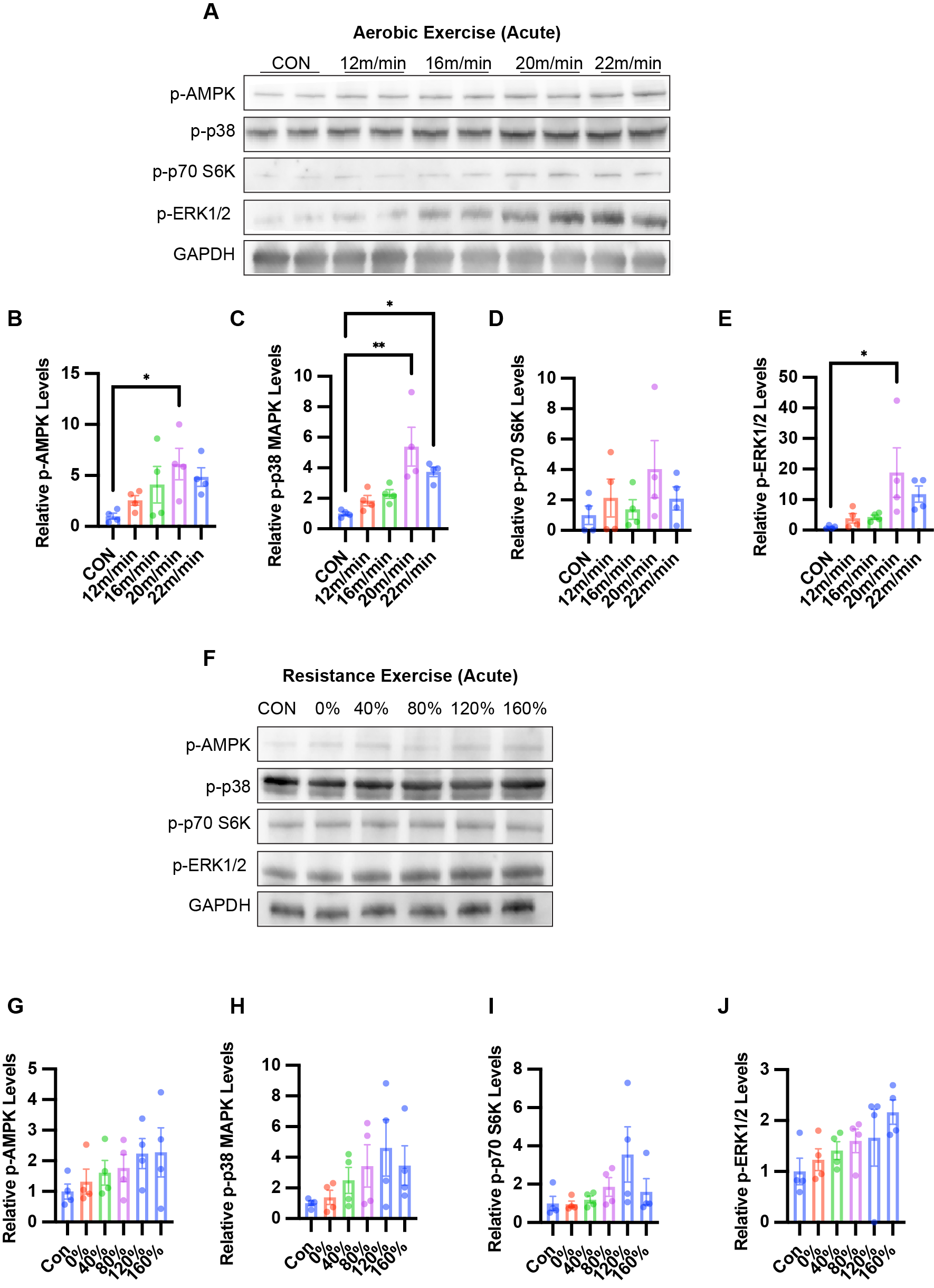
**

**Figure S3. Systematic validation of molecularly defined moderate intensities via signaling kinase profiling.**

(A–E) Representative Western blot images (A) and quantitative analysis of p-AMPK (B), p-p38 MAPK (C), p-p70 S6K (D), and p-ERK1/2 (E) in skeletal muscle following acute aerobic exercise at varying intensities. (F–J) Representative Western blot images (F) and quantitative analysis of p-AMPK (G), p-p38 MAPK (H), p-p70 S6K (I), and p-ERK1/2 (J) in skeletal muscle following acute resistance exercise at varying intensities. GAPDH was used as a loading control. Data are presented as mean ± SEM (n = 4 per group). Statistical significance was determined using one-way ANOVA followed by Tukey’s post-hoc test. *P < 0.05, **P < 0.01 vs. CON.


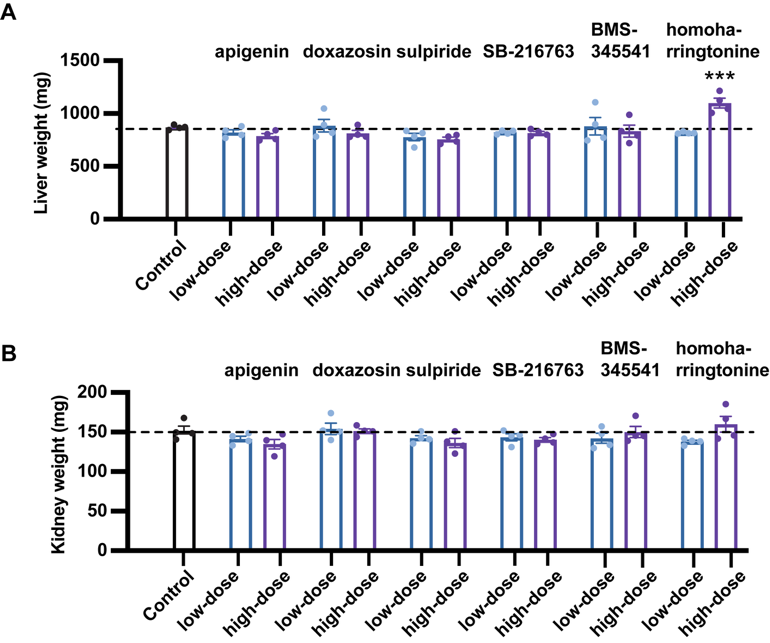


**Figure S4. Effects of compound administration on liver and kidney weights.**

(A) Liver weights following 2 weeks of oral administration of apigenin, doxazosin, sulpiride, SB-216763, BMS-345541, or homoharringtonine, at both low and high doses. (B) Kidney weights under the same treatment conditions. The dashed line represents the mean value of the control group. Data are presented as mean ± SEM. Statistical significance was determined using one-way ANOVA followed by Tukey’s post hoc test. ***P < 0.001 vs. control. n = 4 per group.

**
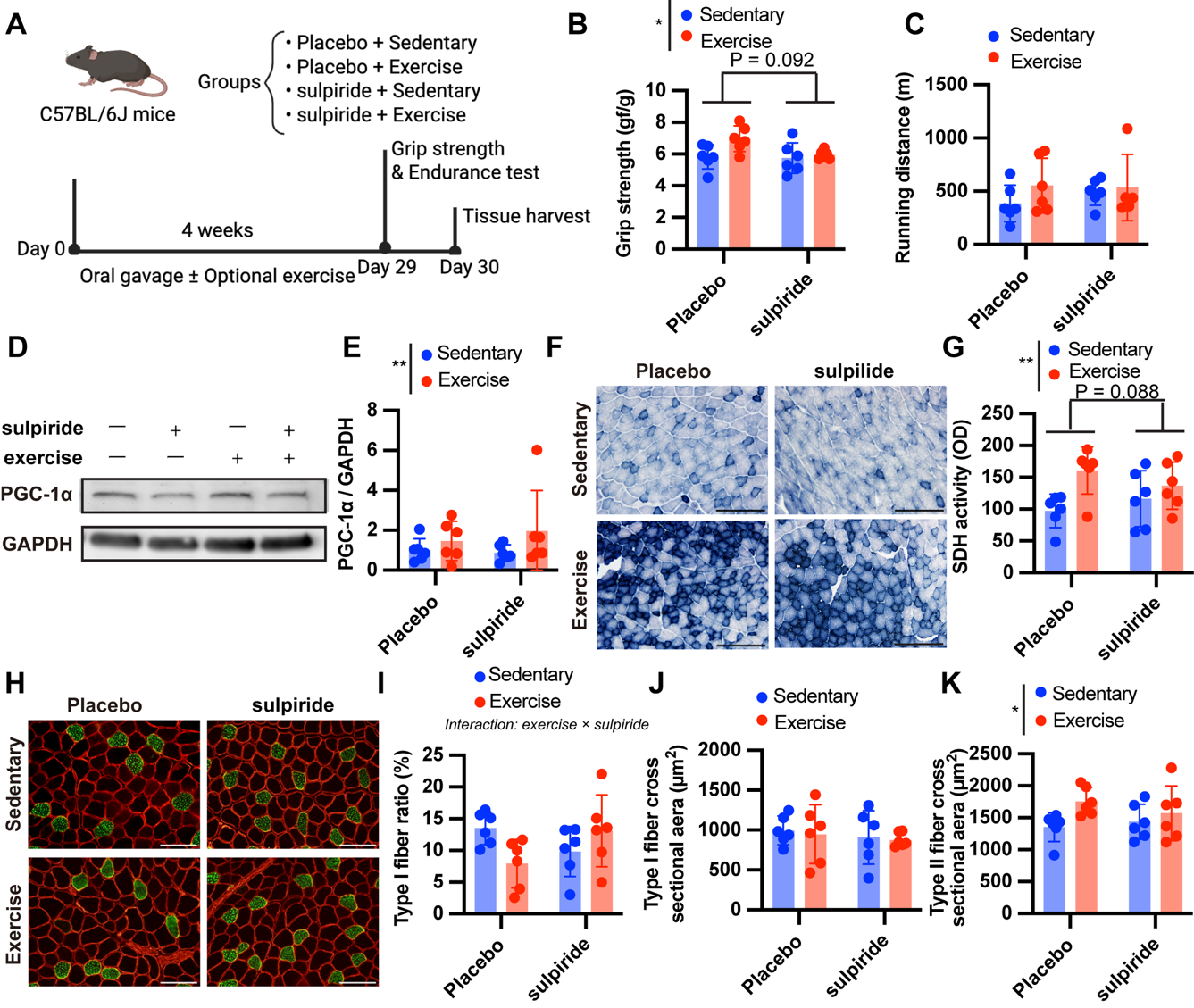
**

**Figure S5. Effects of sulpiride and exercise on muscle performance and adaptation.**

(A) Experimental design. Eight-week-old male C57BL/6J mice were randomly assigned to four groups: Placebo + Sedentary, Placebo + Exercise, sulpiride + Sedentary, and sulpiride + Exercise. Mice received daily oral gavage of sulpiride (20 mg/kg/day) or vehicle for 4 weeks, with or without moderate exercise (treadmill and ladder climbing). Grip strength and endurance tests were performed on day 29, followed by tissue harvest on day 30 (B, C) Motor performance outcomes after 4-week intervention: (B) treadmill running distance to exhaustion and (C) grip strength. (D, E) Protein levels of PGC-1α in the gastrocnemius muscle. (D) Representative Western blot images and (E) quantification normalized to GAPDH. (F, G) SDH activity in gastrocnemius muscle. (F) Representative SDH-stained sections and (G) corresponding quantification. Scale bars, 100 µm. (H–K) Immunohistochemical analysis of muscle fiber composition and size. (H) Representative images showing type I fibers (green, anti–MyHC I) and cell membranes (red, anti-dystrophin) in gastrocnemius muscle. Scale bars, 100 µm. Quantification includes (I) type I fiber ratio, (J) type I fiber cross-sectional area, and (K) type II fiber cross-sectional area. Data are presented as mean ± SEM. Two-way ANOVA was used to assess the main effects of exercise and sulpiride, as well as their interaction. Post hoc comparisons were conducted using Tukey’s test. *P < 0.05, **P < 0.01. A significant interaction was detected only in panel (I). For all other panels, no interaction was observed, and main effects of exercise or sulpiride are reported where applicable. *P < 0.05, **P < 0.01.


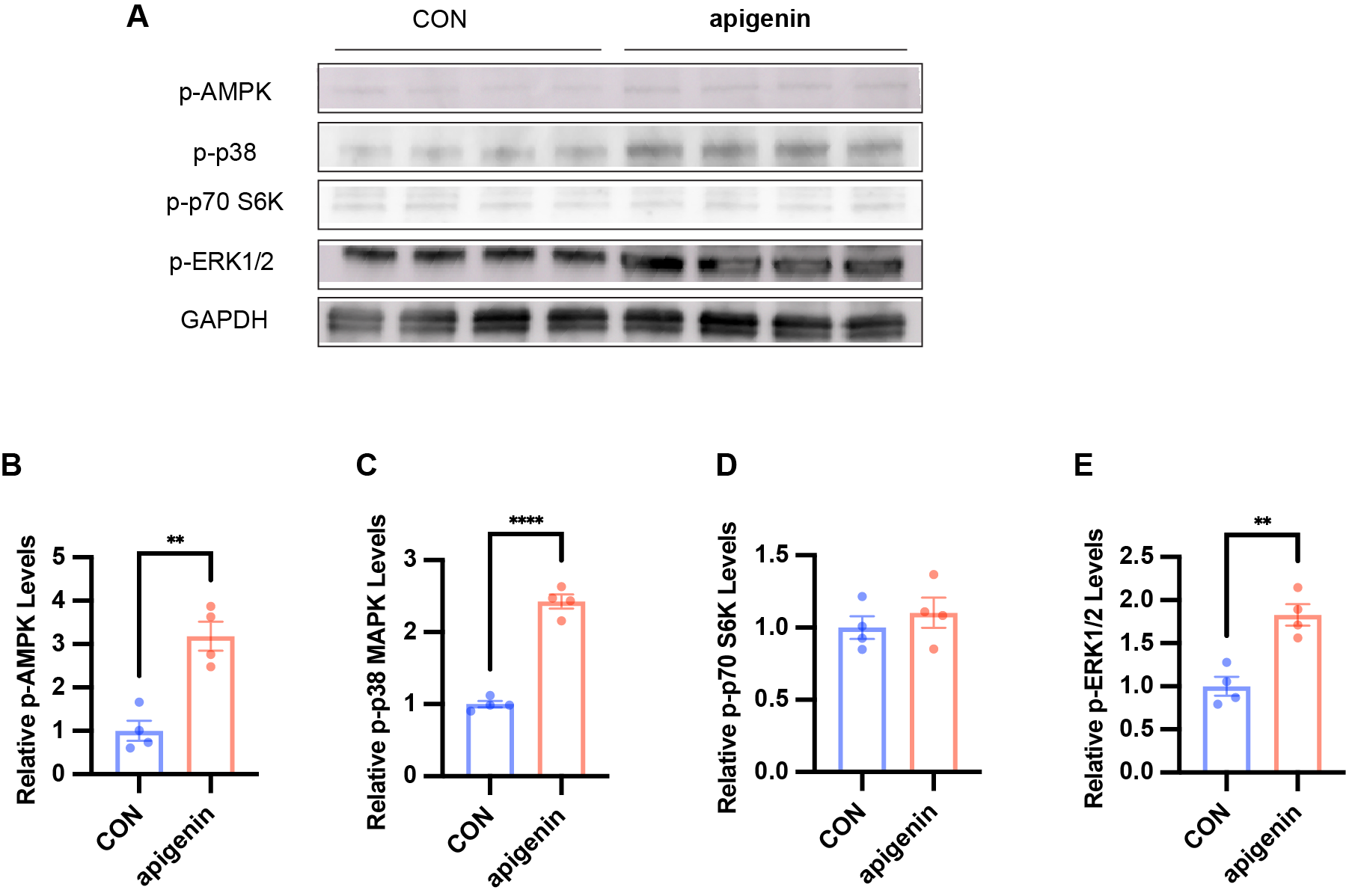


**Figure S6. Apigenin induces broad-spectrum activation of energy-sensing and mechanical adaptive kinase pathways.**

(A) Representative Western blot images of p-AMPK, p-p38 MAPK, p-p70 S6K, and p-ERK1/2 in the skeletal muscle of mice chronically treated with vehicle (CON) or apigenin. (B–E) Quantitative analysis of relative protein phosphorylation levels. GAPDH was used as a loading control. Data are presented as mean ± SEM (n = 4 per group). Statistical significance was determined by unpaired t-test. **P < 0.01, ****P < 0.0001 vs. CON.


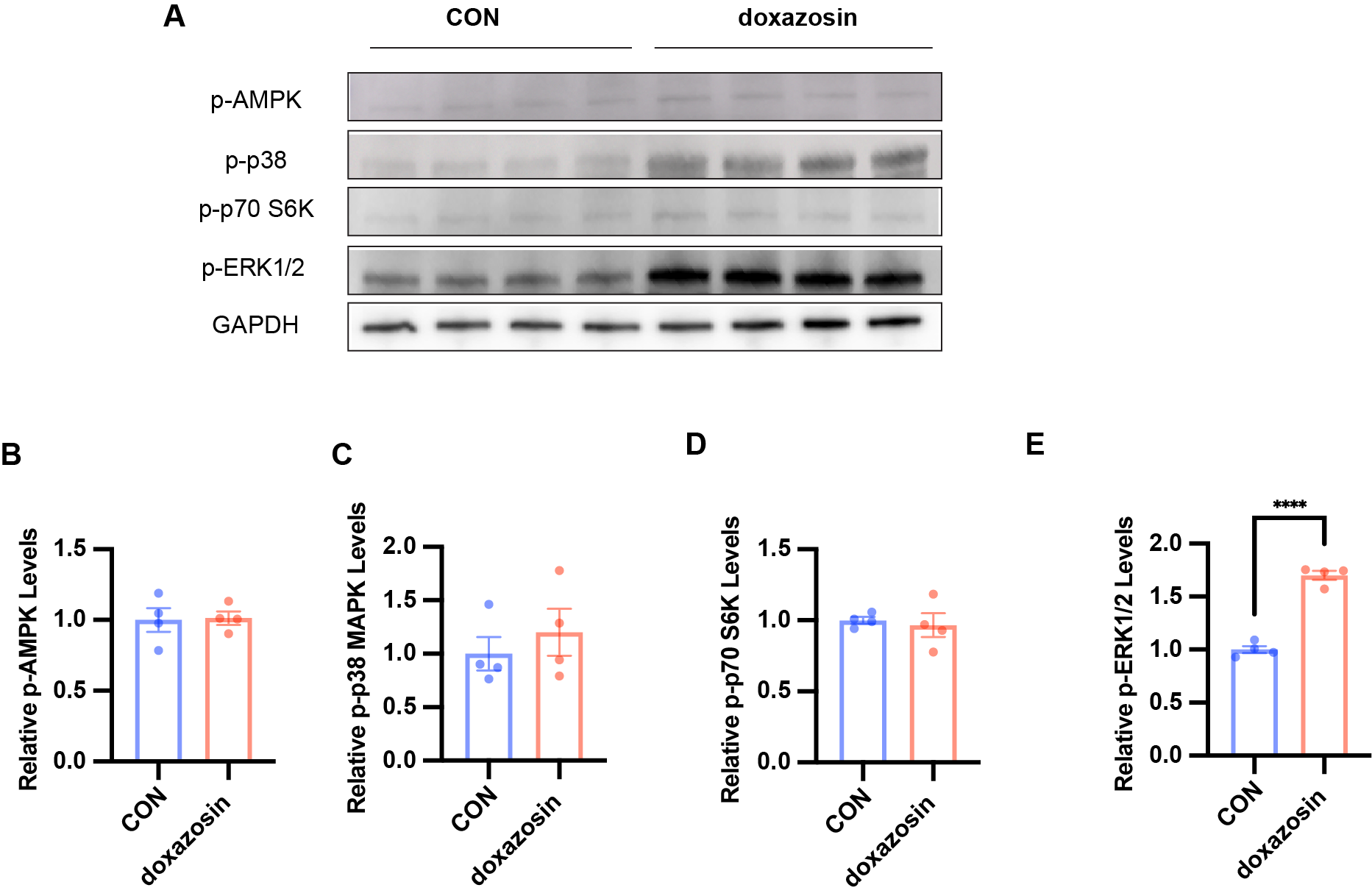


**Figure S7. Doxazosin exerts highly specific activation of the ERK1/2 signaling pathway without altering primary metabolic sensors.**

(A) Representative Western blot images of p-AMPK, p-p38 MAPK, p-p70 S6K, and p-ERK1/2 in the skeletal muscle of mice chronically treated with vehicle (CON) or doxazosin. (B–E) Quantitative analysis of relative protein phosphorylation levels. GAPDH was used as a loading control. Data are presented as mean ± SEM (n = 4 per group). Statistical significance was determined by unpaired t-test. ****P < 0.0001 vs. CON.


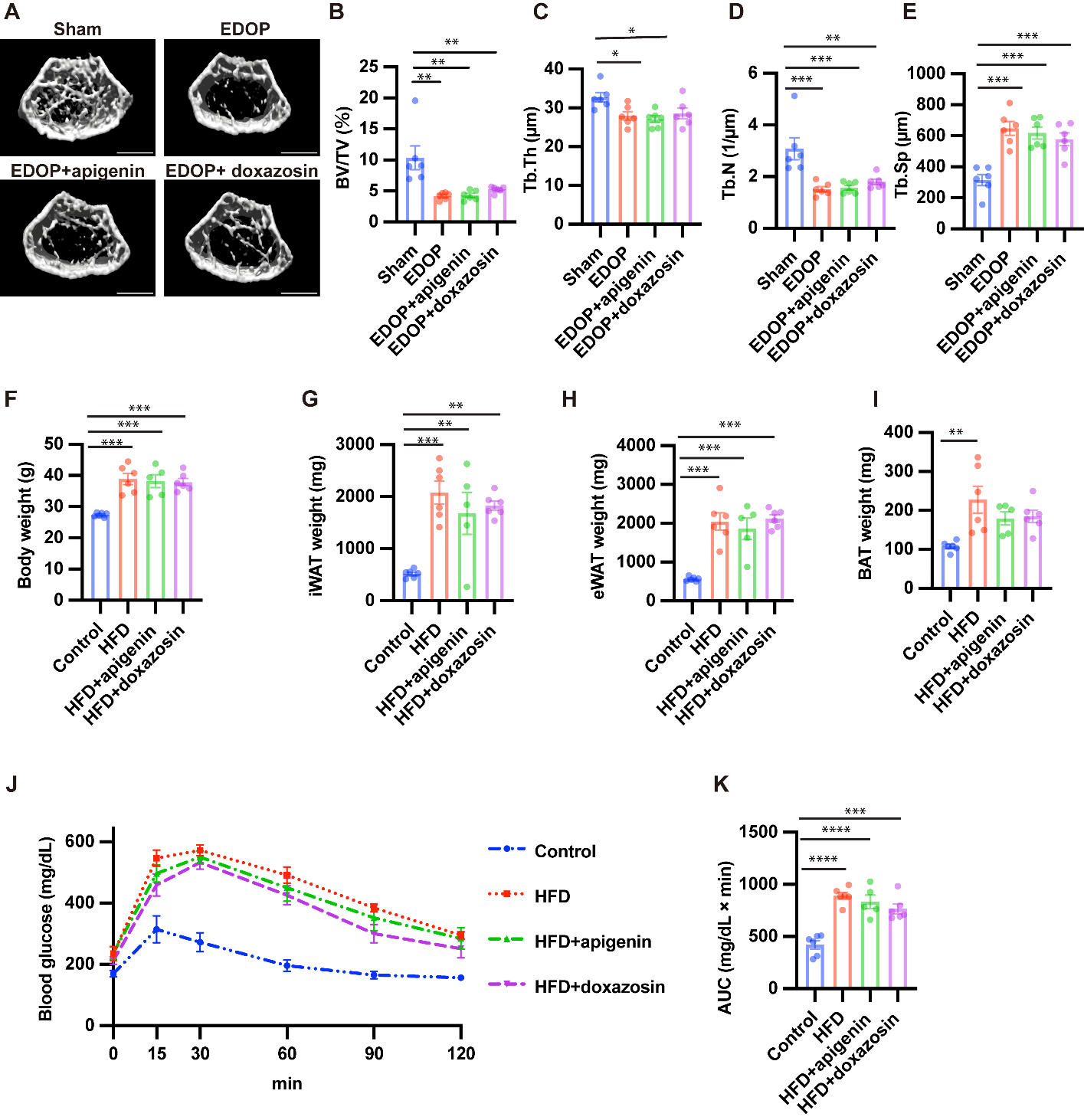
 **Figure S8. Effects of apigenin and doxazosin on bone loss in estrogen deficiency–induced osteoporosis (EDOP) and obesity induced by high-fat diet (HFD).**

(A) Representative μCT images of trabecular bone in the distal femur from mice subjected to EDOP and treated orally with vehicle, apigenin (50 mg/kg/day), or doxazosin (10 mg/kg/day) for 4 weeks. (B–E) Quantitative analysis of trabecular bone parameters: (B) bone volume fraction (BV/TV), (C) trabecular thickness (Tb.Th), (D) trabecular number (Tb.N), and (E) trabecular separation (Tb.Sp). (F–K) Effects of apigenin and doxazosin in mice fed a high-fat diet for 4 weeks (n = 5-6 per group). (F) Body weight. (G–I) Weights of adipose tissues: (G) inguinal white adipose tissue (iWAT), (H) epididymal white adipose tissue (eWAT), and (I) brown adipose tissue (BAT). (J, K) Intraperitoneal glucose tolerance test: (J) time course of blood glucose levels after glucose injection and (K) area under the curve (AUC) of glucose levels. Data are presented as mean ± SEM. Statistical significance was assessed using one-way ANOVA followed by Tukey’s post hoc test. *P < 0.05, **P < 0.01, ***P < 0.001.

**
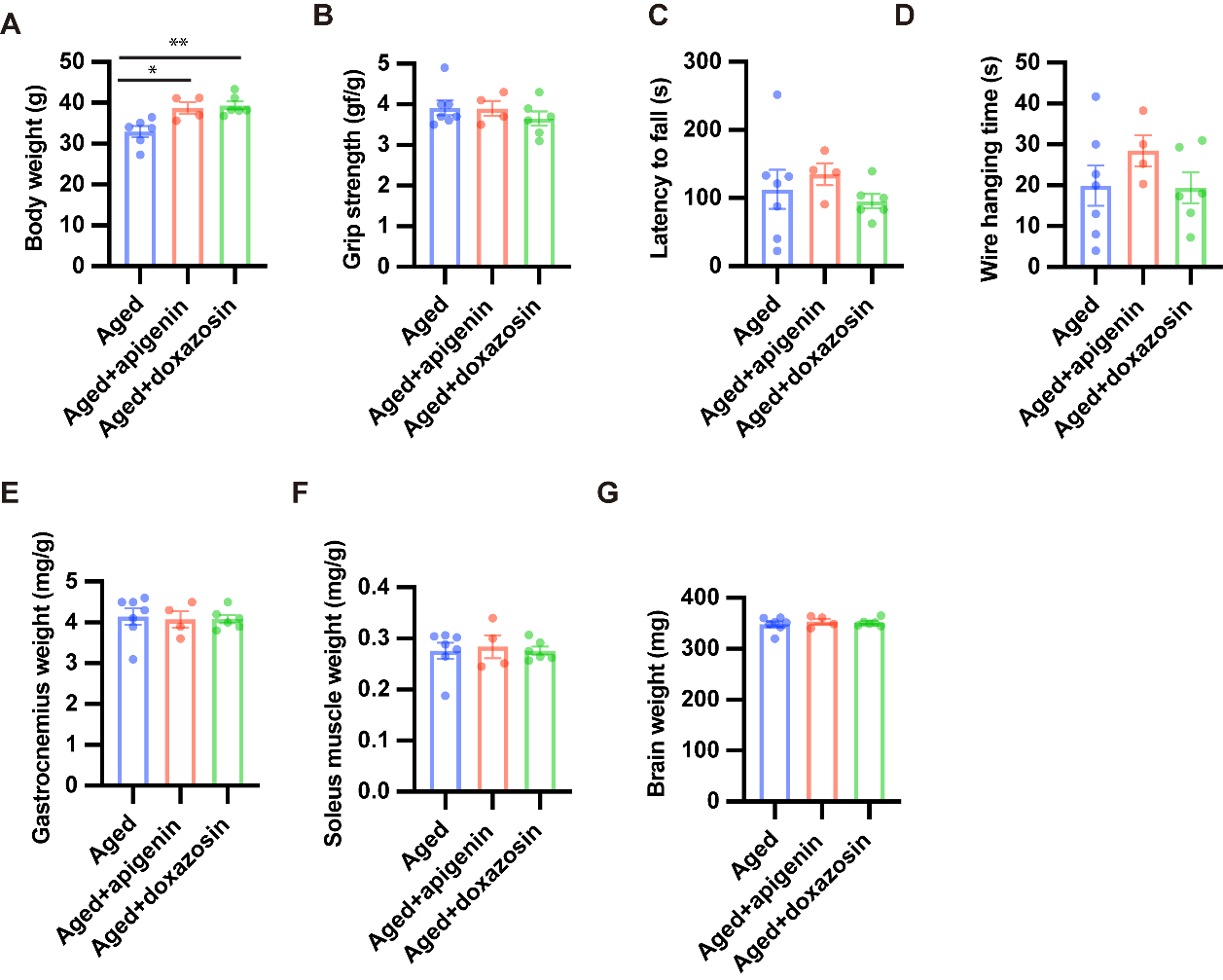
 Figure S9. Effects of apigenin and doxazosin on physical performance and tissue weights in aged mice.**

Aged mice were orally administered vehicle, apigenin (50 mg/kg/day), or doxazosin (10 mg/kg/day) once daily for 4 weeks. (A) Body weight measured at the end of the 4-week treatment period. (B) Forelimb grip strength normalized to body weight. (C) Latency to fall in the rotarod performance test. (D) Hanging time in the wire-hanging test. (E–G) Tissue weights normalized to body weight: (E) gastrocnemius muscle, (F) soleus muscle, and (G) brain weight. Data are presented as mean ± SEM. Statistical significance was determined using one-way ANOVA followed by Tukey’s post hoc test. *P < 0.05, **P < 0.01.


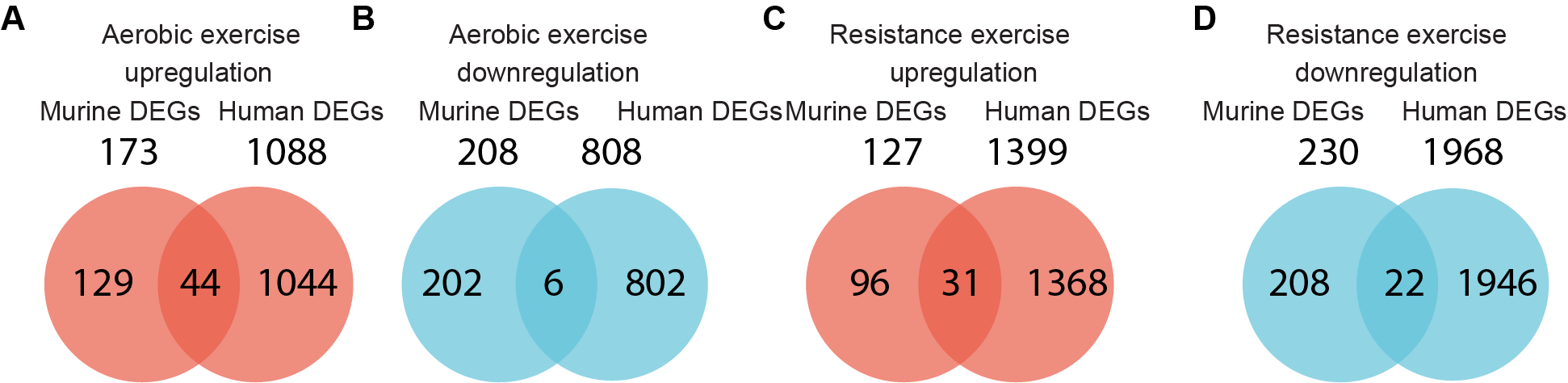


**Figure S10. Cross-species conservation of acute exercise-induced transcriptomic signatures.**

Venn diagrams illustrating the intersection of directionally concordant differentially expressed genes (DEGs) between our murine molecularly defined moderate-intensity exercise model and a comprehensive human skeletal muscle exercise meta-analysis dataset (Pillon et al., 2020). (A, B) Overlapping genes co-upregulated (A; 44 genes) and co-downregulated (B; 6 genes) following acute aerobic exercise. (C, D) Overlapping genes co-upregulated (C; 31 genes) and co-downregulated (D; 22 genes) following acute resistance exercise.

**References**

Pillon, N. J., Gabriel, B. M., Dollet, L., Smith, J. A. B., Sardón Puig, L., Botella, J., Bishop, D. J., Krook, A., & Zierath, J. R. (2020). Transcriptomic profiling of skeletal muscle adaptations to exercise and inactivity. *Nature Communications*, *11*(1), 470. https://doi.org/10.1038/s41467-019-13869-w
